# Prioritizing Molecular Biomarkers in Asthma and Respiratory Allergy Using Systems Biology

**DOI:** 10.3389/fimmu.2021.640791

**Published:** 2021-04-15

**Authors:** Lucía Cremades-Jimeno, María Ángeles de Pedro, María López-Ramos, Joaquín Sastre, Pablo Mínguez, Ignacio Mahillo Fernández, Selene Baos, Blanca Cárdaba

**Affiliations:** ^1^ Immunology Department, IIS-Fundación Jiménez Díaz, Universidad Autónoma de Madrid (UAM), Madrid, Spain; ^2^ Allergy Department, Fundación Jiménez Díaz, Madrid, Spain; ^3^ Center for Biomedical Network of Respiratory Diseases (CIBERES), ISCIII, Madrid, Spain; ^4^ Department of Genetics, IIS-Fundación Jiménez Díaz, UAM, Madrid, Spain; ^5^ Center for Biomedical Network Research on Rare Diseases (CIBERER), ISCIII, Madrid, Spain; ^6^ Biostatistics and Epidemiology Unit, Fundación Jiménez Díaz, Madrid, Spain

**Keywords:** allergy, artificial intelligence, asthma, biomarker, respiratory diseases, systems biology

## Abstract

Highly prevalent respiratory diseases such as asthma and allergy remain a pressing health challenge. Currently, there is an unmet need for precise diagnostic tools capable of predicting the great heterogeneity of these illnesses. In a previous study of 94 asthma/respiratory allergy biomarker candidates, we defined a group of potential biomarkers to distinguish clinical phenotypes (i.e. nonallergic asthma, allergic asthma, respiratory allergy without asthma) and disease severity. Here, we analyze our experimental results using complex algorithmic approaches that establish holistic disease models (systems biology), combining these insights with information available in specialized databases developed worldwide. With this approach, we aim to prioritize the most relevant biomarkers according to their specificity and mechanistic implication with molecular motifs of the diseases. The Therapeutic Performance Mapping System (Anaxomics’ TPMS technology) was used to generate one mathematical model per disease: allergic asthma (AA), non-allergic asthma (NA), and respiratory allergy (RA), defining specific molecular motifs for each. The relationship of our molecular biomarker candidates and each disease was analyzed by artificial neural networks (ANNs) scores. These analyses prioritized molecular biomarkers specific to the diseases and to particular molecular motifs. As a first step, molecular characterization of the pathophysiological processes of AA defined 16 molecular motifs: 2 specific for AA, 2 shared with RA, and 12 shared with NA. Mechanistic analysis showed 17 proteins that were strongly related to AA. Eleven proteins were associated with RA and 16 proteins with NA. Specificity analysis showed that 12 proteins were specific to AA, 7 were specific to RA, and 2 to NA. Finally, a triggering analysis revealed a relevant role for AKT1, STAT1, and MAPK13 in all three conditions and for TLR4 in asthmatic diseases (AA and NA). In conclusion, this study has enabled us to prioritize biomarkers depending on the functionality associated with each disease and with specific molecular motifs, which could improve the definition and usefulness of new molecular biomarkers.

**Graphical Abstract:**
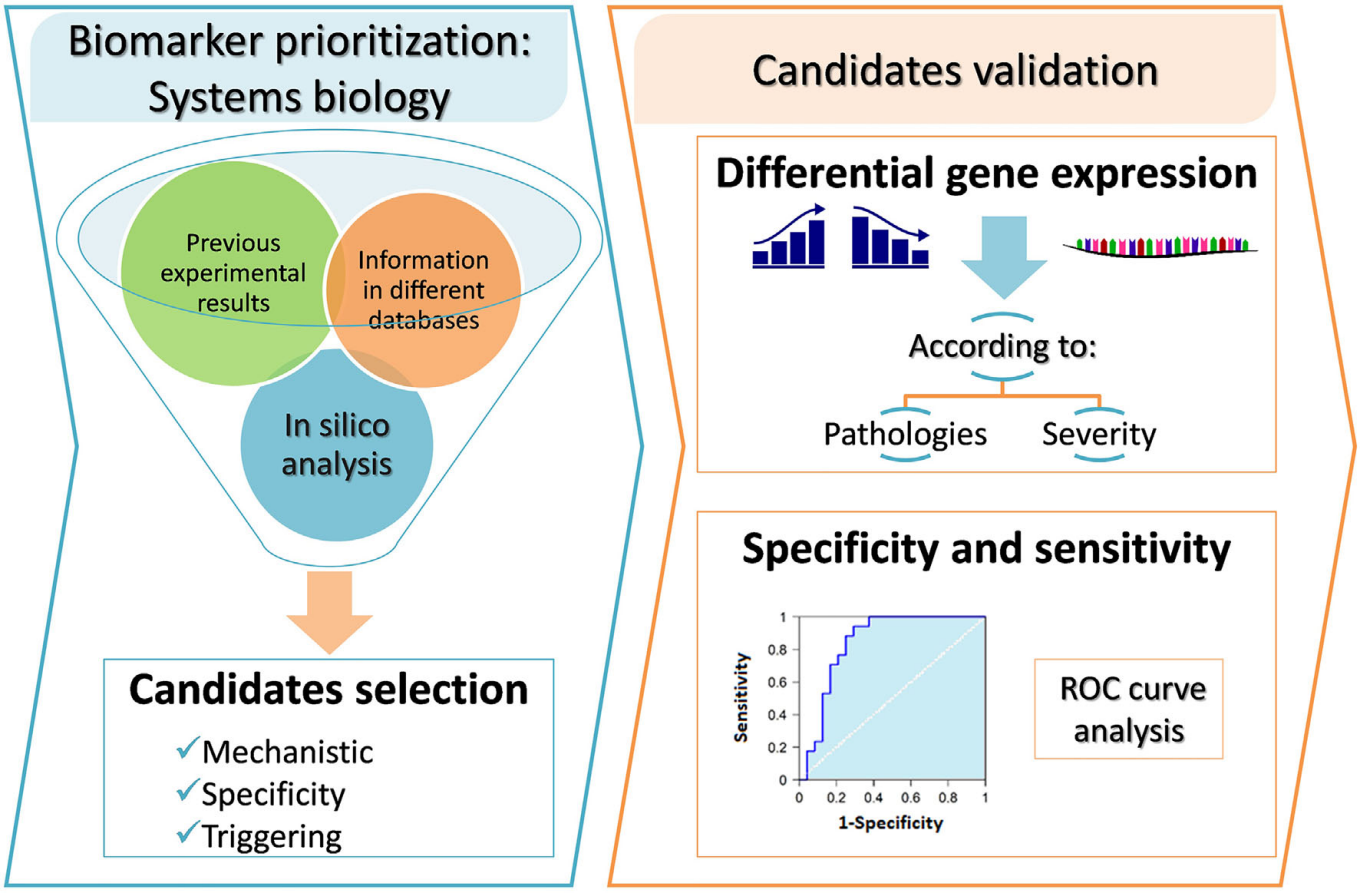


## Introduction

Chronic inflammatory respiratory diseases, including allergic diseases and asthma, are common, complex, and heterogeneous diseases in which the clinical course and treatment response are often challenging to predict. The high prevalence of these diseases and their impact on quality of life make them a serious public-health problem associated with a substantial economic burden.

The World Health Organization (WHO) defines asthma as the most common chronic disease in children, affecting more than 340 million people around the world (1). Approximately 10% of asthmatic patients develop severe clinical symptoms and have significant morbidity and mortality (2). Asthma is a disease that affects the airways, manifesting clinically as cough, dyspnea, chest tightness, shortness of breath, or mucus production. From a clinical point of view, asthma is associated with a progressive reduction in lung function and presence of inflammation. Asthma diagnoses span multiple clinical presentations (phenotypes), each with different pathophysiological mechanisms (endotypes) (3, 4). Allergic mechanisms have been implicated in 50 to 80% of asthmatic patients and in approximately 50% of those with severe disease. Allergic asthma, characterized by an early onset, is the most common asthma phenotype and is traditionally considered as an eosinophilic mediated disease characterized by overexpression and activation of T helper type 2 (Th-2) cells (5). As a result, asthma has been classically associated with type 2 respiratory inflammation, characterized by high levels of IgE, eosinophils, and some cytokines such as IL-4, IL-5, IL-13, and IL-9, canonically associated with allergic responses. However, there are also many clinical conditions where there is a combination of inflammatory cells (eosinophils/neutrophils) and no clear type of respiratory inflammation. Between 10 and 33% of subjects with asthma are not associated with allergy (non-allergic asthma) and exhibit non-type 2 inflammation (non-T2 or T2-low endotype) (6), in many cases with a prevalence of neutrophils; understanding of the immune mechanisms implicated is less developed (7–11).

Advances in our understanding of the immunologic mechanisms implicated in asthma disease have shown that there are many other important players, mainly those related to the innate immune response and its interrelation with tissue cells (derived from epithelial cells), and these are a frequent subject of review (12, 13). Especially relevant was the discovery that type 2 innate lymphoid cells (ILC-2), which have no antigen-specific receptors, could be crucial in mediating airway inflammation in eosinophilic phenotypes of asthma, whether or not these phenotypes are associated with atopic conditions (14). ILC-2 respond to epithelium-derived signals mediated by the so-called “alarmins” [IL-25, IL-33, and thymic stromal lymphopoietin (TSLP)] produced by epithelial cells after injury, through pattern-recognition receptors (15). In fact, allergic asthma, or type 2 immune response, is now considered as a complex network between type 2 cytokines (IL-4, IL-5, IL-9, and IL-13), which are mainly secreted from Th2 cells, IgE-producing B cells, group 2 innate lymphoid cells (ILC-2), and a small fraction of IL-4-producing NK cells and NK-T cells, basophils, eosinophils, mast cells, and alarmins (IL-25, IL-33, and TSLP), which are released from tissue cells, particularly epithelial cells (12).

Despite the clinical complexity of asthma, most efforts to find new treatments have been centered on allergic asthma or asthma mediated by type 2 inflammation. Patients with allergic asthma would be classified under the T2-high endotype. Many targeted treatments (i.e. biological therapy against key elements of T2 inflammatory response such as anti-IL-4/IL-13, anti-IL-4, anti-IL-5, anti-IgE antibodies, anti-CRTH2) are in different stages of clinical development and have produced varying results (16). However, to understand the heterogeneity in T2 inflammation (17), patients with a higher likelihood to respond adequately to this kind of therapies must be identified.

Non-allergic or intrinsic asthma includes a subset of patients with non-T2 inflammation (18, 19). The pathophysiology and exact mechanisms of T2-low (Non-T2) asthma are less thoroughly understood and studied than other asthma types. In general, this type of asthma is characterized by a lack of eosinophilic inflammation/T2 markers and is occasionally associated with neutrophilic or pauci-granulocytic inflammation (20). The major mechanism leading to a non-type 2 response is thought to result from an irregular innate immune response, including intrinsic neutrophil abnormalities and activation of the IL-17-mediated pathway (12). T2-low asthma is common, accounts for one-third to 45% of patients with severe disease, and is associated with poor response to corticosteroid therapy (21). To date, no directed therapy has been found to be effective against this endotype (22).

Thus, there remains an unmet clinical need in the study of the mechanisms and biomarkers for both T2-high and T2-low endotypes as concerns their ability to predict response to targeted therapy (23). Better understanding of the complex immune network of asthma inflammation and key players of immunity are continuously subjected to study (12, 13, 18). Successful therapy of asthma requires better definition of underlying pathogenesis in order to find more precise therapy options or develop methods of precision medicine.

New techniques such as massive analysis or -omics have become important tools in the search for risk-related or protective biomarkers and the development of new drugs (24). Combined with multiple studies derived from -omics technologies, a systems biology approach, which treats disease as a holistic process without any targeted hypothesis, has been fruitfully used in other medical fields in recent years to develop a new molecular medicine (25).

Despite the many advances in this area, there are still large gaps that need to be understood in order to better manage this type of complex diseases, mainly related to the mechanisms involved in each clinical condition. Against this backdrop, many research groups are currently searching for new and complementary molecular biomarkers and respiratory disease endotypes (20, 26). Toward this purpose, we studied the differential gene-expression of 94 biomarker candidates in patients with different clinical respiratory diseases (i.e. respiratory allergy, allergic asthma, non-allergic asthma) (27, 28), defining molecular biomarkers that can discriminate between allergic (T2-high) and non-allergic asthma (T2-low or non-T2) and predict disease severity in non-invasive samples derived from peripheral blood. New genes and protein biomarkers, including CHI3L1, IL-8, IL-10, MSR1, PHLDA1, PI3, and SERPINB2, were proposed to discriminate healthy control subjects from non-allergic asthmatic patients (T2-low) and to determine asthma severity (29). The relevance of those potential biomarkers in asthma and allergy diseases has been discussed extensively (27). Later, we measured the ability of these biomarkers to discriminate between allergic (T2-high) and non-allergic asthma (T2-low or non T2) diseases and determine severity at the gene and protein levels. The results revealed panels of genes and proteins that can discriminate T2-high from T2-low asthma and measure disease severity (30) by using simple techniques and with very good discriminatory parameters. Though encouraging, our initial results showed limitations that should be addressed, and this is the primary motivation of this study. In particular, there were too many genes/proteins considered as “potentially useful,” mainly those related to allergic phenotypes. This is why it was decided to prioritize the role played by these genes/proteins using new approaches as *in silico* studies, which make it possible to combine information from multiple databases with researchers’ own experimental models, thus establishing a model thanks to the use of complex algorithms based on systems biology. With this aim, the three pathophysiological processes of interest (i.e. respiratory allergy, allergic asthma, non-allergic asthma) were defined at the molecular level. Next, the effector proteins of the manifestative and causative motifs of these three highly related processes were characterized at the molecular level, showing common and differential molecular motifs for these pathologies. This study aims to classify and define the usefulness of sets of biomarkers in order to provide additional diagnostic and therapeutic functional-targeted tools for these types of diseases.

## Materials and Methods

### Molecular Characterization of Respiratory Allergy, Allergic Asthma, and Non-Allergic Asthma

Firstly, the molecular characterization of the three pathophysiological processes of interest (respiratory allergy, allergic asthma, and non-allergic asthma) was performed using the Therapeutic Performance Mapping System (TPMS) technology (Anaxomics Biotech, Barcelona, Catalonia, Spain) (31). Briefly, systems biology generates models that are able to reproduce the behavior of a disease in a patient, thus identifying the key genes, proteins, or metabolites in the development of the disease. A dictionary has been created to translate clinical and medical terms into molecular biology data, effectively linking the molecular and the clinical words. This dictionary, called the Biological Effectors Database (BED), relates biological processes (adverse events of drugs, drug indications, diseases, etc.) with the proteins most closely associated with them. Thus, the dictionary acts as a translator of clinical phenotypes into terms comprehensible for protein networks, and conversely allows for the translation of molecular measures toward clinical outcomes. The BED is structured hierarchically, where the biggest level is the entire disease, which is divided into different pathophysiological molecular motifs, which in turn contain the proteins involved in the development of the disease. The motifs are classified into two levels depending on their respective implication, i.e. causal motifs, which are directly related to the onset or pathophysiology of the condition, and symptomatic (manifestative) motifs, which are a consequence of the disease.

**Figure 1:**
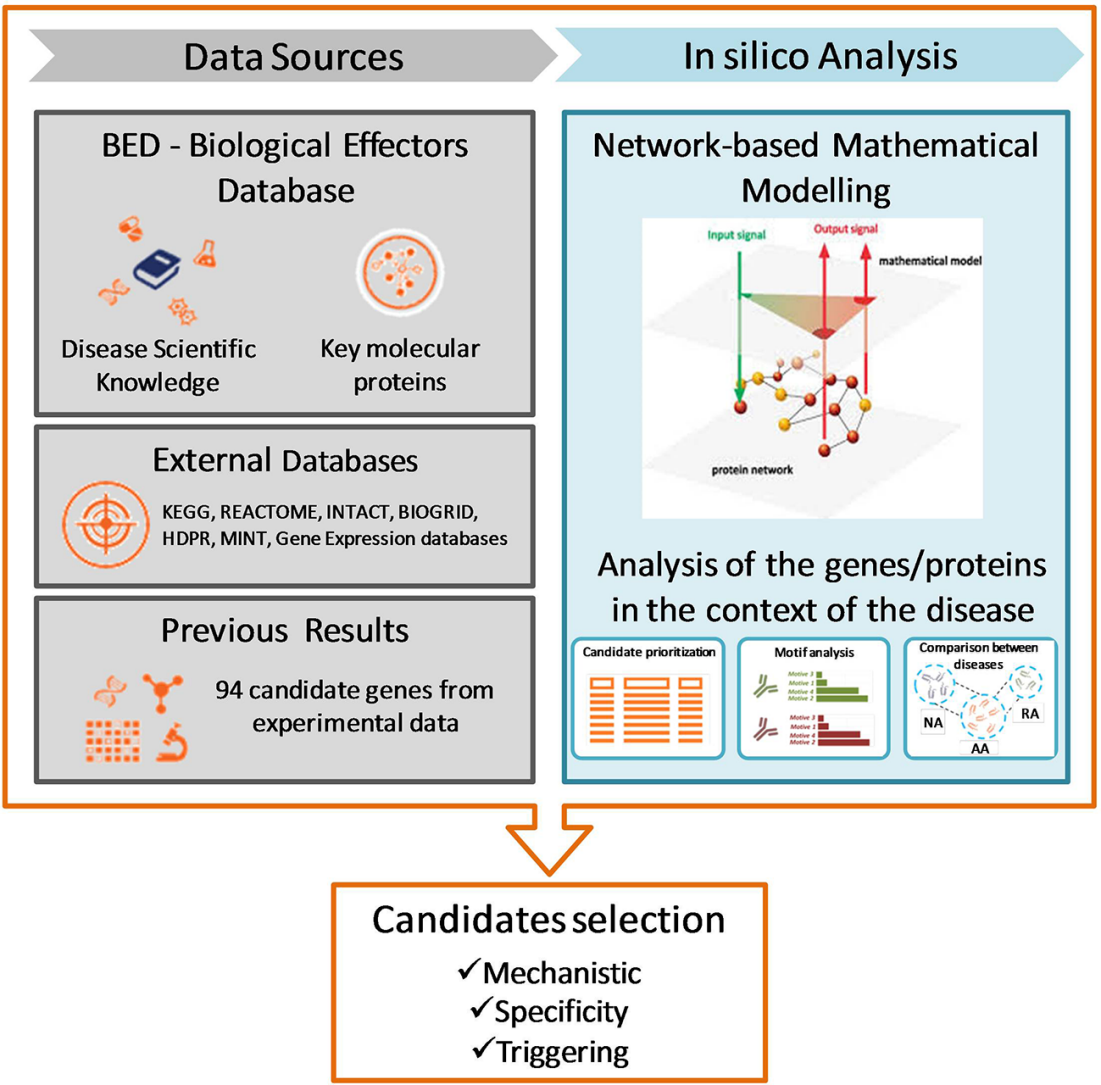
Study design and workflow of systems biology study. NA, non-allergic Asthma; AA, Allergic Asthma; RA, Respiratory Allergy. [Based on Gimenez et al. (32)].

In the present study, respiratory allergy, allergic asthma, and non-allergic asthma have been characterized at the molecular level. Therefore, the analysis of high throughput data by means of TPMS allows for identification of those proteins closely associated with the disease of interest and can provide a mechanistic rationale for their involvement. The effector proteins of the manifestative and causal molecular motifs of these three diseases have been identified through bibliographic review and curate data. **Figure 1** summarizes the workflow used for this study.

### Artificial Neural Network (ANN) Analysis: Biomarker Prioritization

#### Mathematical Model Generation

To create mathematical models, it was builded a biological map around the key disease proteins defined during the characterization (**Figure 1**). The map was extended by adding knowledge-oriented connectivity layers, i.e., protein-to-protein interactions, including physical interactions and modulations, signaling, metabolic relationships, and gene expression regulation. Data were obtained from the following public and private external databases: KEGG (33), REACTOME (34), INTACT (35), BIOGRID (36), HDPR (37), and MINT (38) in addition to the scientific literature reviewed. At the same time, the network is embedded with all sorts of biological information derived from public sources (e.g. drug targets, tissue expression, biomarkers) about nodes (i.e. proteins) and edges (i.e. connections).

The 94 biomarker candidate proteins previously studied by our group (27, 28) were ranked according to their mechanistic relationship to each of the conditions of interest by means of artificial neural networks (ANNs) as indicated by TPMS technology (31).

Then, the models were trained with a proprietary Truth Table containing public and our list of candidates (28) related to each disease. This acts as a set of conditions that must be fulfilled to resemble the situation under study. Here, we have used artificial intelligence (AI) technologies to model complex network behavior, including graph theory and statistical pattern recognition technologies; genetic algorithms; artificial neural networks; dimensionality reduction techniques; and stochastic methods such as Monte Carlo simulated annealing, among others. ANNs identify relations among regions of the network (generalization). TPMS technology uses proprietary stochastic optimization algorithms based on ANNs to identify plausible protein interaction networks learning from those sets of prior knowledge. Specifically, the ANNs applied (39) use relationships between drug targets and clinical elements (BED) mapped onto the human proteome network compiled in a hand-screened truth table [based on the information contained in DrugBank about drugs and indications (40) for training a classifier]. The accuracy with which ANNs reproduce the indications of DrugBank is 98% for compounds with all targets in the human biological network after applying a cross-fold validation process (41–44). The models thus obtained reproduce the average behavior of patients under evaluation in agreement with current scientific understandings, allowing effective, mechanistically driven analyses.

#### Analysis of Proteomic Data in the Context of the Selected Disease

The 94 candidate genes proposed by our previous studies (27, 28) were compiled and processed in a format readable by TPMS technology. By means of artificial neural networks (ANN), the estimated effect of changes to these proteins on the selected disease was assessed. The system attempts to find the shortest distance between candidates and the effectors of the disease, thus generating a list of potential biomarkers ordered by their association with the selected disease. Finally, after checking the proteins that passed this automatic filter, the final candidates were selected in accordance with the published literature (**Figure 1**). ANN analyses score the proteins according to their predicted mechanistic relationships with the effector proteins of the molecular characterization of respiratory allergy, allergic asthma, and non-allergic asthma. ANN score is a numerical value that indicates the percentage of relationship between 2 sets of proteins (0–100%). Here, one set comprised each of our candidate proteins and the other was the set that defines the disease or its molecular motifs.

Two kinds of analyses were performed: the analysis of the relationship of each one of the candidate proteins with each of the entire disease or whole pathology (which provides an idea of how each protein relates to all the proteins involved in the disease); and analysis according to molecular motifs. The disease could be divided into several motifs that do not necessarily have to be closely related from a topological point of view. A protein could be topologically related to one motif with a low number of proteins that is not topologically related to the rest of proteins involved in the disease and, thus, the protein appears as weakly related to the characterization of the whole disease. Thus, a motif analysis can elucidate relationships between candidate proteins and the disease (considered as a group of motifs) that an analysis considering the entire disease would not reveal.

The use of ANNs permitted mechanistic ranking of the target proteins according to their relationship with each whole disease and with each molecular motif individually. Four categories were used to group the analyzed proteins according to the predicted relationship value: strongly related proteins (“*Very high*” group, ≥92% predicted ANN value, p < 0.01); highly related proteins (“*High*” group, with a predicted ANN value <92–≥78%, p values between 0.01 and 0.05); moderately related proteins (“*Medium*,” with a predicted ANN value <78–≥38, *P* value between 0.05 and 0.25); and proteins with a mild relationship (“*Low*” group,<38% predicted ANN value, *P* value >0.25).

The final ranking was performed using two measures based on the relationship of each protein with whole diseases and the specific motifs:

- Relationship level: indicates the level of relationship of the proteins with each overall disease or any disease-specific motif.
- Specificity: indicates whether the protein presents a stronger relationship with the specific motifs of the disease than with the specific motifs of the other diseases.

### Validation of Prioritized Biomarkers

The most useful (specific) biomarkers defined theoretically by systems biology were contrasted with our previous data of gene expression analyzed by RT-qPCR in RNA extracted from peripheral blood mononuclear cells (PBMCs) in a population of 114 subjects made up of healthy control subjects, respiratory allergic patients, allergic asthmatic subjects, and non-allergic asthmatic patients (27, 28). Significance of gene expression was defined by RQ (relative quantification) defined previously (27, 28).

The sensitivity and specificity of the best molecular biomarkers defined by systems biology was determined by ROC curve analysis in our study population (27, 28). A ROC curve was constructed for the candidate biomarkers common with our previous results and the defined by systems biology (biomarkers that showed high relationship to at least one of the studied condition). Eighteen kinds of comparisons were performed to determine the most effective biomarkers to distinguish among the different clinical conditions: healthy control group, non-allergic asthmatic patients, allergic asthmatic patients, and patients with respiratory allergy without asthma, as well as different severities in asthma groups. Details of the population studied were described previously (27). A guide for interpreting the ROC curves has been described previously (29). Only those results with a 95% confidence interval (95% CI) of between 0.70 and 1 were considered as statistically significant.

### Triggering Analysis

Finally, this method based on systems biology was also used to analyze the ability of each biomarker candidate protein to cause the activation of the effector proteins that define respiratory allergy, allergic asthma, and non-allergic asthma. The objective of this strategy was to identify the proteins of interest whose modulation can promote the activation of the highest number of proteins involved (coverage) in these three diseases. It is an analysis that evaluates the ability of a set of genes to activate the disease. The results reflect which proteins, together, are capable of activating the highest percentage of disease-defining proteins. The possible role of the proteins of interest as respiratory allergy, allergic asthma, and non-allergic asthma triggers was also assessed.

Two types of scores were obtained:

Individual probability score: this value ranks the proteins of a given set according to their individual probability to act as triggers, that is, how likely a single protein is to act as a trigger. The probability within each protein set ranges from 0 to 1. It is represented in an approximate asterisk scale, in which the proteins with the highest probability have up to five asterisks assigned, and the ones with the lowest probability have only one asterisk.

Cumulative score: indicates the percentage of effectors of the condition triggered by the alteration of a protein together with the rest of potential triggering, i.e. the cumulative score shows the added effect of combining the evaluated protein with all the previously evaluated proteins.

### Statistical Analysis

The PPI network was visualized using Cytoscape V3.6.1 (https://cytoscape.org/). The relevant hub-genes were screened using the node degrees calculated in Cytoscape. Also, analyses of pathway interconnection between triggering and specific proteins and/or mechanistic proteins for each disease were analyzed by Pathlinkers application from Cytoscape.

The levels and relative expression of the proteins studied were compared between groups by unpaired t-test, using the Graph-Pad InStat 3 program. Statistical significance was established at a two-tailed P value <0.05. ROC curve analyses were performed using the R program.

## Results

### Molecular Motifs of Diseases

**Table 1** summarizes the common and differential molecular motifs that make up the diseases. A total number of 16 molecular motifs have been characterized, which, in combination, are representative of the three conditions of interest.

- Respiratory allergy: composed of two molecular motifs (*acute response* and *late-phase response*, highlighted in blue in **Table 1**), also present in allergic asthma.
- Allergic asthma: composed of 16 molecular motifs. All the motifs are shared with respiratory allergy or non-allergic asthma, with the exception of *Th2-mediated pulmonary inflammation* and *goblet cell hyperplasia* which are specific to allergic asthma, and *granulocyte (eosinophil) infiltration*, which show a stronger implication in allergic than in non-allergic asthma (in lilac in **Table 1**).
- Non-allergic asthma: composed of 12 molecular motifs, all shared with allergic asthma. However, some present higher specificity for nonallergic asthma than for allergic asthma (*Th17-mediated pulmonary inflammation*, and *neutrophil infiltration*, pink in **Table 1**), whereas one of them has a stronger implication in allergic than in non-allergic asthma [*granulocyte (eosinophil) infiltration*, in lilac in **Table 1**].

**Table 1:** Summary of the molecular motifs characterized for respiratory allergy, allergic asthma, and non-allergic asthma.

Specific Molecular Motif	Respiratory Allergy	Allergic Asthma	Non-allergic Asthma
Acute Response	•	•	X
Late-Phase Response	•	•	X
Th2-Mediated Pulmonary Inflammation	X	•	X
Goblet Cell Hyperplasia	X	•	X
Granulocyte (eosinophil) Infiltration	X	•	Δ
Th17-Mediated Pulmonary Inflammation	X	Δ	•
Neutrophil Infiltration	X	Δ	•
Dendritic Cell Activation	X	•	•
ECM Deposition	X	•	•
Angiogenesis Asthma	X	•	•
Airway Smooth Muscle Hypertrophy/Hyperplasia	X	•	•
Epithelial Dysfunction	X	•	•
Airway Smooth Muscle Hypercontractibility	X	•	•
Innervation And Hyper-Excitability	X	•	•
Bronchoconstriction	X	•	•
Mucus Production	X	•	•

• Strong implication of the molecular motif in the disease. Δ Weaker implication compared to the other variant of asthma, though still relevant. X No implication of the molecular motif in the disease. The strongest differential molecular motifs for each disease are indicated in color (Blue: respiratory allergy, Lilac: allergic asthma, Pink: non-allergic asthma).

### Biomarker Prioritization

To obtain the ranking of the best candidate proteins identified as relevant in respiratory allergy, allergic asthma, and non-allergic asthma according to their mechanistic relationship with each of these diseases, three kinds of analyses were performed: ranking of the proteins according to their mechanistic relationship to the diseases of interest, ranking according to their associated pathophysiological motifs, and assessment of the specificity of the candidate proteins to each disease (addressing, especially, the most specific motifs of each disease).

Proteins presenting many interactions, or proteins that do not have reported interactions, were not included in the network as they could disrupt the correct assessment of existing functional relationships. Therefore, one protein (C3AR1) out of the 94 proteins of interest was excluded from the analysis as it is not included in the network used.

#### Individual Evaluation

The mechanistic ranking by ANN analysis allows for classification of the list of 94 proteins based on their predicted functional or mechanistic relationship with a given condition. Here, 19 independent ANNs were carried out, one for each condition (respiratory allergy, allergic asthma, and non-allergic asthma) and one for each condition-specific molecular motif.

##### Protein Predicted Ranking to Entire Disease

The list of all proteins analyzed and the ANN score or relationship predicted values to the entire disease are presented in **Supplementary Table 1**. Whether the proteins are effectors of the disease is also displayed.

**Table 2** summarizes the stronger predicted relationship for each disease. A total number of 11 proteins presented a “high” or stronger predicted relationship with respiratory allergy (**Table 2A**). All of them were effector proteins. For allergic asthma, 17 proteins reached a “high” relationship level (**Table 2B**), one of which (BAX) was not an effector of the disease. Finally, for non-allergic asthma, 16 proteins reached a “high” relationship level with the disease (**Table 2C**) and all were identified as effectors of the disease.

Table 2Summary of proteins presenting a “HIGH” or stronger relationship level with each disease.

**Table:** 

A. Respiratory Allergy			
**Uniprot ID**	**Gene name**	**Respiratory Allergy ANN score**	**Reference**
P05113	***IL5***	**92.43**	Gani F et al. (45)
P35225	***IL13***	91.82	Gani F et al. (45)
P22301	***IL10***	90.26	Urry Z et al. (46)
P05231	***IL6***	90.15	Rose-John S et al. (47)
P15248	***IL9***	89.85	Urry Z et al. (46)
P01579	***IFNG***	88.99	Urry Z et al. (46)
P05112	***IL4***	88.49	Gani F et al. (45)
P43116	***PTGER2***	88.16	Nagai H (48)
P60568	***IL2***	88.13	Schwarz M et al. (49)
P24394	***IL4R***	85.21	Keegan AD et al. (50)
P14784	***IL2RB***	84.15	Mulloy JC et al. (51)

**B. Allergic Asthma**			
**Uniprot ID**	**Gene name**	**Allergic asthma ANN score**	**Reference**
P98088	***MUC5AC***	88.43	Evans CM et al. (60)
P09917	***ALOX5***	87.92	Nagata M et al. (70)
P36222	***CHI3L1***	86.53	Pniewska E et al. (64)
Q8N138	***ORMDL3***	83.15	Loxham M et al. (63)
P12724	***RNASE3***	82.96	Lacy P et al. (62)
Q15063	***POSTN***	82.79	Heijink IH et al. (58)
A8K7I4	***CLCA1***	82.78	Woodruff PG et al. (57)
Q9HC84	***MUC5B***	82.64	Evans CM et al. (60)
P24394	***IL4R***	81.40	Evans CM et al. (60)
Q16552	***IL17A***	81.22	Chakir J et al. (54)
O95760	***IL33***	81.12	Davies DE (52)
P14151	***SELL***	81.04	Nadi E et al. (71)
Q9H293	***IL25***	80.90	Whelan T et al. (59)
P05120	***SERPINB2***	80.82	Woodruff PG et al. (57)
P27930	***IL1R2***	79.06	Knolle MD et al. (72)
Q07812	***BAX***	78.36	–
P01137	***TGFB1***	78.21	Yalcin AD et al. (73)

**C. Non-allergic Asthma**			
**Uniprot ID**	**Gene name**	**Nonallergic Asthma**	**Reference**
P98088	***MUC5AC***	91.14	Evans CM et al. (60)
P36222	***CHI3L1***	89.91	Pniewska E et al. (64)
Q15063	***POSTN***	89.75	Heijink IH et al. (58)
O95760	***IL33***	89.01	Davies DE (52)
P12724	***RNASE3***	88.08	Lacy P et al. (62)
P14151	***SELL***	88.01	Nadi E et al. (71)
Q8N138	***ORMDL3***	87.70	Loxham M et al. (63)
Q9HC84	***MUC5B***	86.88	Evans CM et al. (60)
P09917	***ALOX5***	86.69	Nagata M et al. (70)
Q16552	***IL17A***	86.69	Chakir J et al. (54)
P43116	***PTGER2***	84.79	Nagai H (48)
P13501	***CCL5***	83.26	Isgrò M et al. (74)
Q9H293	***IL25***	82.81	Whelan T et al. (59)
A8K7I4	***CLCA1***	81.62	Woodruff PG et al. (57)
P05120	***SERPINB2***	79.93	Woodruff PG et al. (57)
P51671	***CCL11***	78.30	Isgrò M et al. (74)

ANN score is defined in methods. Four categories were used to group the analyzed proteins according to the predicted relationship value; here we represent the two main associated categories for each disease analyzed: Strongly related proteins (“Very high” group, ≥92% predicted ANN value, associated P-value <0.01) and Highly related proteins (“High” group, with a predicted ANN value <92–≥78%, associated P-value 0.01–0.05). References correspond to the definition of the respective protein as an effector protein. Strongly related proteins are labeled in bold. Blue: Respiratory Allergy, Lilac: Allergic Asthma, Pink: Non-Allergic Asthma.

**Figure 2:**
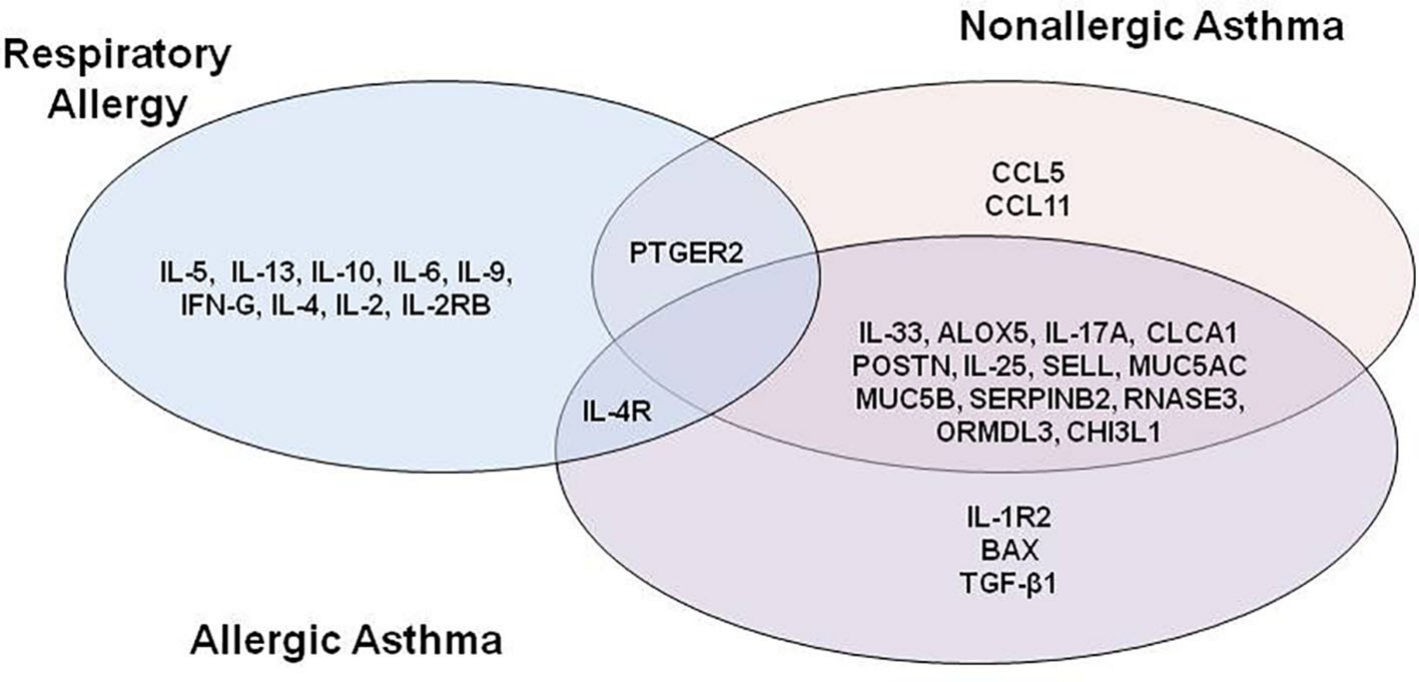
Venn diagram of proteins with “high” or stronger relationship level with at least one disease.

Looking at the overlap of the proteins with a “high” or stronger relationship level (**Figure 2**), two results are worth mentioning: there were no proteins with a “high” or stronger relationship value with the three conditions at the same time, and the highest number of overlapping proteins (n = 13) was found between non-allergic and allergic asthma conditions, all of which were effectors of shared molecular motifs (**Table 3**).

**Table 3:** Summary of commom proteins with a “high” or stronger relationship with allergic and non-allergic asthma.

Uniprot ID	Gene name	Molecular motif	Reference
**O95760**	***IL33***	Dendritic Cell Activation, Angiogenesis Asthma	Davies DE (52); Guo Z et al. (53)
**P09917**	***ALOX5***	Granulocyte Infiltration	Guo Z et al. (53)
**Q16552**	***IL17A***	Granulocyte Infiltration, Th17-Mediated Pulmonary Inflammation, Mucus Production	Chakir J et al. (54); Chesné J et al. (55); Barnes PJ (56)
**A8K7I4**	***CLCA1***	Airway Smooth Muscle Hypercontractibility	Woodruff PG et al. (57)
**Q15063**	***POSTN***	Granulocyte Infiltration, Angiogenesis Asthma	Heijink IH et al. (58); Woodruff PG et al. (57)
**Q9H293**	***IL25***	Angiogenesis Asthma	Woodruff PG et al. (57)
**P14151**	***SELL***	Granulocyte Infiltration	Whelan T et al. (59)
**P98088**	***MUC5AC***	Airway Smooth Muscle Hypercontractibility, Mucus Production	Evans CM et al. (60); Qi L et al. (61)
**Q9HC84**	***MUC5B***	Airway Smooth Muscle Hypercontractibility	Qi L et al. (61)
**P05120**	***SERPINB2***	Granulocyte Infiltration	Woodruff PG et al. (57)
**P12724**	***RNASE3***	Granulocyte Infiltration	Lacy P et al. (62)
**Q8N138**	***ORMDL3***	ECM Deposition	Loxham M et al. (63)
**P36222**	***CHI3L1***	Angiongenesis Asthma	Pniewska E et al. (64)

The molecular motifs for which these proteins act as effectors and the corresponding bibliographic reference are also displayed.

Respiratory allergy and allergic asthma share 1 highly related protein, specifically IL-4R, which has been identified as an effector of the acute phase of allergy (60) and receptor of IL-4 and IL-13, molecules that have been proven to regulate IgE synthesis (45) and have been detected to be over-expressed in acute asthmatics.

Interestingly, PTGER2, a prostaglandin receptor with functions in both the acute phase allergy molecular motif (48) and bronchoconstriction asthma and allergic asthma molecular motif, although being reported as playing a role in the three diseases, presented a highly functional relationship to respiratory allergy and non-allergic asthma, but a moderate relationship to allergic asthma.

##### Individual Molecular Motifs Relationship Analysis

Next, to understand the implication of the proteins in the diseases studied, an independent ANN analysis was performed for each molecular motif defined for the three pathological conditions (respiratory allergy, allergic asthma, and non-allergic asthma) (**Table 4**). Two molecular motifs (*acute response* and *granulocyte infiltration*) presented proteins with a “very high” relationship level. All molecular motifs presented at least one protein with a “high” relationship level with the exception of *goblet cell hyperplasia*, *Th17-mediated pulmonary inflammation*, *ECM deposition*, and *innervation and hyper-excitability*. All the proteins predicted to have a “high” or stronger relationship level with a given molecular motif were identified as effectors of the molecular motif.

**Table 4:** Ranking of Proteins classified by association with molecular motifs (Effectors proteins are underlined).

Specific Molecular Motif	Very High	HIGH	MEDIUM
Acute Response	**IL-10, IL-4, IL-2, IL-9, IFNG, PTGER2**	**IL-4R, IL-2RB**	**IL-13, IL-5, FOXP3, IL-6, NFATC1, TLR4, IL-1R1, TNF, TGFB1, STAT1, MAPK13, IL-1R2, IL-33, ZAP70, TSLP, NFKBIZ, CD40, CD48, LYN, NLRP3, ADRB1.**
Late-Phase Response	**-**	**IL-13, IL-5, IL-6, TNF**	**IL-10, IL-4, FOXP3, NFATC1, IL-2, IL-4R, TNFAIL3, STAT1, MAPK13, IL-9, IFNG, TSLP, FPR3, ALOX5.**
Th2-Mediated Pulmonary Inflammation	**-**	**IL-10, IL-13, IL-4, IL-5, CCL17**	**FOXP3, IL-6, NFATC1, IL-2, IL-4R, TNFAIL3, TLR4, BAX, IL-1R1, CCL11, AKT1, TNF, TGFB1, STAT1, MAPK13, IL-9, IFNG, CCL5, IL-1R2, IL-33, ZAP70.**
Goblet Cell Hyperplasia	**-**	**-**	**IL-5, FOXP3, IL-6, NFATC1, IL-2, TNFAIP3, TLR4, TGFB1, MAPK13, IFNG, CCL5, ZAP70, IL-2RB, PTPRC, SPP1, SOS1, DUSP1, SVIL, LGALS3.**
Granulocyte (eosinophil) Infiltration	**INFG**	**IL-4R, CCL11, TGFB1, CCL5, CD40, RNASE3, ALOX5, SELL, IL-17A, ITGAL, POSTN, SERPINB2**	**IL-6, NFATC1, TLR4, TNF, STAT1, MAPK13, PTPRC, VCAN, SPP1, FPR3, NOS2, EIF5A, S1PR5, SMURF1, LYN, NCF2, ALOX15, GPX3.**
Th17-Mediated Pulmonary Inflammation	**-**	**-**	**IL-10, FOXP3, IL-6, NFATC1, TNF, TGFB1, STAT1, MAPK13, CD40, CD86, IL-17A, NLRP3, IL-25.**
Neutrophil Infiltration	**-**	**IL-6, LGALS3**	**FOXP3, NFATC1, TNFAIP3, TLR4, BAX, IL-1R1, AKT1, TNF, TGFB1, STAT1, MAPK13, CCL5, IL-1R2, IL-8, FPR3, CTSC, NFKBIZ, APAF1, NOS2, S100A9, S1PR5, NLRP3, NCF2**
Dendritic Cell Activation	**-**	**TLR4**	**NFATC1, IL-1R1, AKT1, TGFB1, MAPK13, IL-1R2, IL-33, TSLP, NFKBIZ, S100A9, HLA-DQB1, HLA-DRB1, IL-25.**
ECM Deposition	**-**	**-**	**NFATC1, TNFAIP3, TLR4, BAX, AKT1, TNF, TGFB1, STAT1, MAPK13, IFNG, CCL5, ZAP70, PTPRC, SPP1, FPR3, CD40, SOS1, NOS2, CD86, SVIL, RNASE3, ORMDL3, S100A9, LGALS3, NCF2, ITGB7, ITGB8.**
Angiogenesis_Asthma	**-**	**IL-33, ADAM33, CHI3L1, POSTN**	**NFATC1, TLR4, TNF, TGFB1, STAT1, MAPK13, VCAN, NOS2, NCF2.**
Airway Smooth Muscle Hypertrophy/Hyperplasia	**-**	**TNF**	**IL-5, CCL17, FOXP3, IL-6, NFATC1, IL-2, TNFAIP3, TLR4, IL-1R1, CCL11, AKT1, TGFB1, STAT1, MAPK13, IFNG, CCL5, IL-1R2, ZAP70, IL-2RB, IL-8, VCAN, SPP1, FPR3, CTSC, NFKBIZ, DUSP1, NOS2, S100A9, IRAK3, NCF2.**
Epithelial Dysfunction	**-**	**CCL5**	**FOXP3, IL-6, NFATC1, TNFAIP3, TLR4, CCL11, TNF, TGFB1, STAT1, MAPK13, IFNG, VCAN, SPP1, FPR3, CD86, CTSG.**
Airway Smooth Muscle Hypercontractibility	**-**	**IL-4R, IL-1R2, MUC5AC**	**IL-10, IL-13, IL-4, IL-5, IL-6, IL-2, STAT1, IL-9, IFNG, TSLP, CD40, NOS2, CLCA1, MUC2, MUC5B.**
Innervation And Hyper-Excitability	**-**	**-**	**ZAP70, PTPRC, DUSP1, CD86.**
Bronchoconstriction	**-**	**PTGER2, ADRB1**	**IL-8, CLCA1.**
Mucus Production	**-**	**IL-8**	**IL-13, CCL17, NFATC1, CCL11, MAPK13, IL-9, CCL5, SPP1, FPR3, SOS1, S1PR5, IL-17A, MUC5AC.**

These results classified the proteins by their specific relationship with molecular motifs. These results were taken into account for the final specificity analysis.

##### Specificity Analysis: Protein Ranking

Finally, to maximize the specificities of the proteins of interest, information from entire diseases and molecular motif analysis were combined taking into account the results obtained in the molecular motifs of differential weight for each disease:

- Respiratory Allergy: *allergy*, *acute response*, and *late-phase response* ANN results were combined.
- Allergic asthma: *Allergic asthma*, *Th2-mediated pulmonary inflammation*, *goblet cell hyperplasia*, and *granulocyte (eosinophil) infiltration* ANN results were combined.
- Non-allergic Asthma: *Asthma*, *Th-17 mediated pulmonary inflammation*, and *neutrophil inflammation* ANN results were combined.

Two different parameters (relationship level and specificity) were evaluated as explained in the methods section above. The results are summarized in **Table 5**.

**Table 5:** Specificity ranking of the proteins with a high relationship to at least one of the studied conditions (overall disease and/or molecular motifs).

A. Respiratory Allergy								
Protein information			Conditions					
			Respiratory Allergy					
Uniprot ID	Gene name	In topology	Respiratory Allergy ANN score	Specific Motifs ANN score		Relationship level	Specific motifs relationship	
				Acute response	Late-phase response			
P24394	*IL4R*	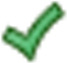	85.21	83.48	39.30	High	High	
P05113	*IL5*	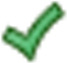	92.43	72.65	81.36	Very high	High	
**P43116**	***PTGER2***	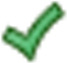	**88.16**	**92.44**	**5.58**	**Very high**	**Very high**	
P35225	*IL13*	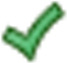	91.82	72.16	81.03	High	High	
**P22301**	***IL10***	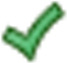	**90.26**	**94.51**	**75.18**	**Very high**	**Very high**	
P05231	*IL6*	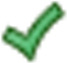	90.15	70.66	83.91	High	High	
**P01375**	***TNF***	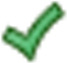	**75.99**	**76.33**	**83.71**	**High**	**High**	
**P15248**	***IL9***	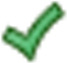	**89.85**	**93.02**	**75.72**	**Very high**	**Very high**	
P01579	*IFNG*	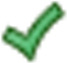	88.99	94.00	71.68	Very high	Very high	
**P05112**	***IL4***	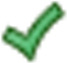	**88.49**	**93.03**	**75.72**	**Very high**	**Very high**	
**P60568**	***IL2***	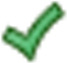	**88.13**	**93.90**	**70.14**	**Very high**	**Very high**	
**P14784**	***IL2RB***	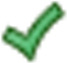	**84.15**	**87.59**	**29.56**	**High**	**High**	

B. Allergic Asthma								
Protein information			Conditions					
			Allergic Asthma					
Uniprot ID	Gene name	In topology	Allergic Asthma ANN score	Specific Motifs ANN score			Relationship level	Specific motifs relationship
				Th2-mediated inflammation	Goblet cell hyperplasia	Granulocyte infiltration		
P98088	*MUC5AC*	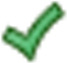	88.43	9.46	10.10	8.51	High	Low
P36222	*CHI3L1*	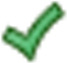	86.53	10.50	7.97	9.76	High	Low
**Q15063**	***POSTN***	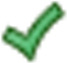	**82.79**	**10.33**	**9.32**	**85.87**	**High**	**High**
O95760	*IL33*	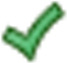	81.12	39.98	9.77	10.24	High	Medium
**P12724**	***RNASE3***	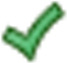	**82.96**	**15.95**	**8.81**	**83.89**	**High**	**High**
**P14151**	***SELL***	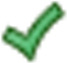	**81.04**	**12.94**	**11.97**	**88.36**	**High**	**High**
Q8N138	*ORMDL3*	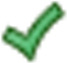	83.15	15.95	12.64	7.61	High	Low
Q9HC84	*MUC5B*	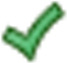	82.64	9.46	10.10	8.51	High	Low
P24394	*IL4R*	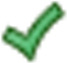	81.40	64.45	33.49	83.51	High	High
**P09917**	***ALOX5***	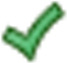	**87.92**	**15.19**	**13.17**	**91.42**	**High**	**High**
P05113	*IL5*	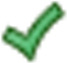	71.80	80.56	49.32	19.41	High	High
**Q16552**	***IL17A***	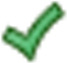	**81.22**	**11.68**	**8.86**	**85.81**	**High**	**High**
**P13501**	***CCL5***	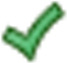	**77.01**	**41.77**	**45.46**	**89.83**	**High**	**High**
Q9H293	*IL25*	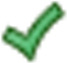	80.90	8.59	8.80	7.03	High	Low
A8K7I4	*CLCA1*	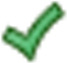	82.78	14.41	9.99	15.48	High	Low
**P25942**	***CD40***	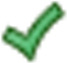	**74.19**	**18.84**	**18.05**	**78.62**	**High**	**High**
**P20701**	***ITGAL***	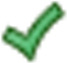	**77.00**	**11.30**	**10.84**	**81.65**	**High**	**High**
**P05120**	***SERPINB2***	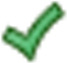	**80.82**	**7.98**	**22.20**	**90.37**	**High**	**High**
P35225	*IL13*	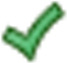	71.19	85.95	9.02	26.72	High	High
P22301	*IL10*	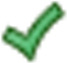	72.16	86.08	16.31	24.93	High	High
P27930	*IL1R2*	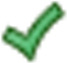	79.06	40.74	29.16	12.68	High	Medium
P01579	*IFNG*	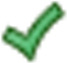	71.11	42.66	49.77	92.43	Very high	Very high
P05112	*IL4*	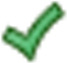	69.62	83.34	12.53	32.52	High	High
**P51671**	***CCL11***	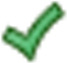	**75.25**	**56.74**	**10.05**	**88.73**	**High**	**High**
Q07812	*BAX*	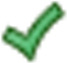	78.36	61.27	16.58	23.43	High	Medium
**P01137**	***TGFB1***	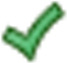	**78.21**	**53.84**	**45.86**	**91.04**	**High**	**High**
**Q92583**	***CCL17***	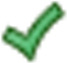	**77.87**	**79.66**	**11.87**	**37.72**	**High**	**High**

C. Non-allergic Asthma								
Protein information			**Conditions**					
			Non-allergic Asthma					
Uniprot ID	Gene name	In topology	Non-allergic Asthma ANN score	Specific Motifs ANN score		Relationship level	Specific motifs relationship	
				Th17-mediated inflammation	Neutrophil infiltration			
P98088	*MUC5AC*	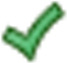	91.14	10.58	7.59	High	Low	
P36222	*CHI3L1*	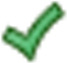	89.91	16.37	10.88	High	Low	
Q15063	*POSTN*	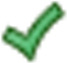	89.75	12.03	11.19	High	Low	
O95760	*IL33*	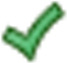	89.01	9.40	13.22	High	Low	
P12724	*RNASE3*	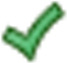	88.08	12.22	13.19	High	Low	
P14151	*SELL*	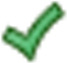	88.01	15.73	10.55	High	Low	
Q8N138	*ORMDL3*	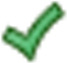	87.70	11.79	11.85	High	Low	
Q9HC84	*MUC5B*	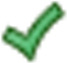	86.88	10.58	7.59	High	Low	
P09917	*ALOX5*	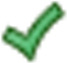	86.69	7.91	14.47	High	Low	
Q16552	*IL17A*	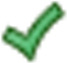	86,69	75,50	10,62	High	Medium	
P43116	*PTGER2*	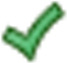	84,79	12,22	11,92	High	Low	
P13501	*CCL5*	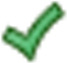	83,26	17,66	45,33	High	Medium	
**Q9H293**	***IL25***	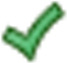	**82,81**	**69,62**	**8,22**	**High**	**Medium**	
A8K7I4	*CLCA1*	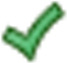	81,62	15,94	15,13	High	Low	
**P17931**	***LGALS3***	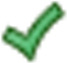	**77,78**	**16,32**	**80,96**	**High**	**High**	
P05120	*SERPINB2*	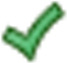	79,93	15,73	16,07	High	Low	
P05231	*IL6*	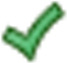	74,83	48,44	80,97	High	High	
P51671	*CCL11*	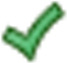	78,30	10,58	11,85	High	Low	

Specific proteins according to the criteria defined in *Methods* are remarked in bold. The final ranking was performed using two measures Relationship level and Specificity. Relationship level: indicates the level of relationship of the proteins with each overall disease or any disease-specific motif. Specificity: indicates whether the protein presents a stronger relationship with the specific motifs of the disease than with the specific motifs of the other diseases.

The specificity ranking led to the identification of seven allergy specific proteins (**Table 5A**). The relationship found can be partially explained by the inflammatory component of the molecular motifs of allergy. In fact, the most strongly related proteins are directly involved in inflammatory processes (e.g. IL-2, IL-2RB, TNF, or PTGER2) or in the regulation of these processes (IL-10, IL-4, and IL-9).

A total number of 12 proteins have been identified to be specifically related to allergic asthma (**Table 5B**): ALOX5, RNASE3, TGFB1, CCL5, ITGAL CD40, SERPINB2, CCL11, POSTN, IL-17A, CCL17, and SELL. Among these, only CCL17 displayed a stronger non-specific relationship with allergic asthma when compared to the value obtained for the asthma evaluation.

Finally, two proteins were identified as more closely related to non-allergic asthma (**Table 5C**) according to specificity analysis, namely IL-25 and LGALS3. Moreover, LGALS3 was the only one to display a stronger overall relationship with nonallergic than with allergic asthma.

In addition, some proteins that showed no specific relationship with any condition did present a distinct high overall relationship with them. Specifically, IFN-G, IL-13, IL-5, and IL-4R showed an especially high overall relationship with allergic conditions (respiratory allergy and allergic asthma).

MUC5B, ORMDL3, MUC5AC, CHI3L1, CLCA1, and IL-33 display a distinctly high non-specific relationship with allergic and non-allergic asthma.

### Validation of Prioritized Results by Systems Biology

**Figure 3:**
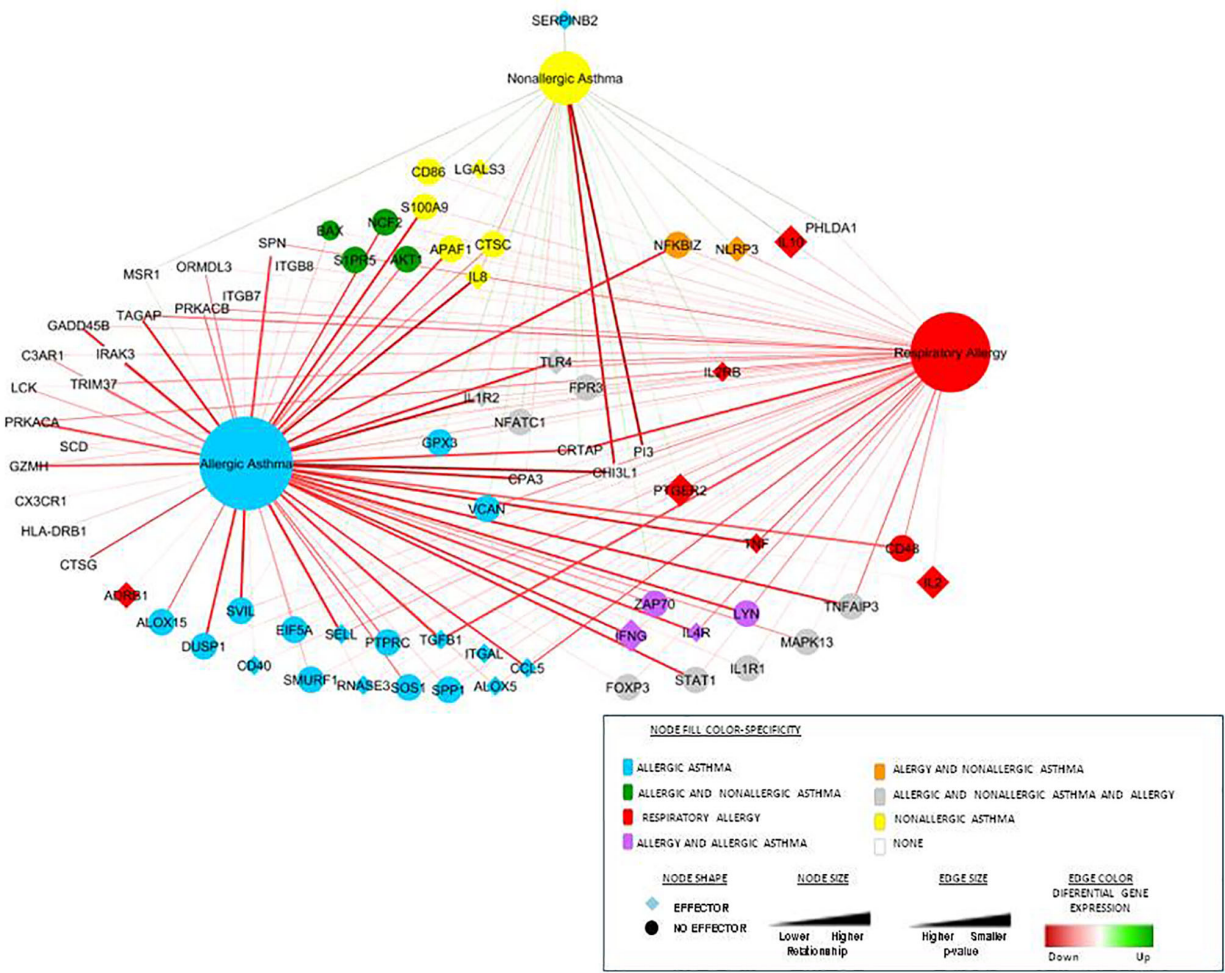
Representation of the three diseases and their connections combined with the differential gene-expression proteins obtained from our experimental data. This network includes connections between the diseases (the three main nodes) and the most differential gene-expression proteins obtained from our experimental data (27, 28). The node shape illustrates whether the protein is an effector or not, color represents specificity and size and the level of relationship. Differential gene expression is represented by the edge color and the *p*-value by the size.

To validate the results of specificity obtained by systems biology, we compared our previous gene-expression data and constructed an ROC curve to analyze the experimental gene-expression data by disease condition. **Figure 3** summarizes the graphical overview of the three diseases combining the results of systems biology analysis of the total 94 biomarkers in each disease and our experimental data of gene-expression by clinical comparisons against healthy control subjects (RQ value), obtaining an overview of the diseases nodes interactions. This graph (**Figure 3**) combines the mathematical model results with our gene-expression results, showing differential and shared nodes for each disease with mechanistic information (effector proteins are indicated).

Next, the gene-expression analysis of each specific protein defined by systems biology was checked in order to compare the theoretical results and experimental data. **Table 6** summarizes the gene expression data for each specific protein defined per disease. Overall, there was a correlation between theoretical specificity and experimental results, although several of the “specific biomarkers” determined by systems biology were not classified as specific by our results despite the fact that these were relevant for the disease compared with healthy controls (because were relevant to more than one of the diseases). Several proteins were not compared due to a lack of experimental data, as indicated in the footnotes of each Table.

Table 6Relative Quantification (RQ) values of gene expression in specific proteins defined by systems biology.

**Table:** 

A. Respiratory Allergy (RA) specific proteins						
Gene	RA *vs* Control		AA *vs* Control		NA *vs* Control	
	RQ	adjusted P	RQ	adjusted P	RQ	adjusted P
***IL2RB***	0.21	3.79E-8	0.38	9.24E-8	2.68	3.5E-4
***TNF***	0.13	3.5E-10	0.066	3.47E-21	0.41	0.011
***PTGER2***	0.30	7.3E-8	0.48	1.36E-6	1.86	0.003
***IL10***	0.15	5.06E-6	nd	nd	6.14	1.6E-6

No data for IL-4, IL-9, and IL-2. nd, not determined.

**Table:** 

B. Allergic Asthma (AA) specific proteins						
Gene	RA *vs* Control		AA *vs* Control		NA *vs* Control	
	RQ	adjusted P	RQ	adjusted P	RQ	adjusted P
***ALOX5***	0.25	2.8E-5	0.23	6.13E-8	nd	nd
***RNASE3***	0.41	8.9E-3	0.35	6.09E-4	nd	nd
***TGFB1***	0.20	3.5E-13	0.18	4.7E-20	nd	nd
***CCL5***	0.13	2.8E-10	0.14	3.18E-12	nd	nd
***ITGAL***	0.39	5.3E-6	0.45	2.58E-5	nd	nd
***CD40***	0.26	7.2E-5	0.35	3.48E-5	nd	nd
***SERPINB2***	nd	nd	1.67	nd	8.94	7.6E-5
***SELL***	0.14	4.25E-8	0.10	3.2E-15	0.53	0.039

No data for CCL11, POSTN, IL17A, CCL17.

**Table:** 

C. Non-allergic Asthma (NA) specific proteins						
Gene	RA *vs* Control		AA *vs* Control		NA *vs* Control	
	RQ	adjusted P	RQ	adjusted P	RQ	adjusted P
***LGALS3***	0.46	9.9E-4	0.46	1.9E-5	2.06	0.006

No data for IL-25.

Finally, to determine the specificity and sensitivity of the biomarkers defined by systems biology (biomarkers that showed high relationship to at least one of the studied condition), ROC curve analysis was performed using our experimental results to establish their ability to determine diseases and severities. There were 13 common biomarkers (*ALOX5*, *CCL5*, *CHI3L1*, *IFNG*, *IL10*, *IL1R2*, *IL4R*, *IL8*, *SELL*, *SERPINB2*, *TGFB1*, *TLR4*, *TNF*) defined as potential good candidates by our previous experimental data (27, 28) and by the system biology analysis. The best biomarkers (AUC > 0.75) by clinical conditions are summarized in **Table 7**.

**Table 7:** ROC analysis of gene expression in the 13 common proteins defined by systems biology and experimental data.

A. Comparison between healthy controls and diseases				
Control *vs.* Respiratory Allergy				
Gene	N (C)	N (RA)	AUC (95% CI)	Threshold
***IL1R2***	27	14	0.82 (0.67–0.96)	8.91
***IL4R***	27	14	0.79 (0.65–0.94)	9.9
***SELL***	27	14	0.78 (0.63–0.93)	9.04
***TLR4***	27	14	0.86 (0.75–0.97)	9.9
***CCL5***	27	14	0.87 (0.75–0.98)	6.11
***TGFB1***	28	14	0.77 (0.59–0.96)	6.92

Control *vs.* Allergic asthma										
Gene	N (C)	All severities			Severe asthma			Moderate-Mild asthma		
		N (AA)	AUC (95% CI)	Threshold	N (S-AA)	AUC (95% CI)	Threshold	N (MM-AA)	AUC (95% CI)	Threshold
***ALOX5***	27	30	0.79 (0.66–0.91)	8.28	15	0.78 (0.63–0.93)	7.96	15	0.79 (0.65–0.93)	8.3
***CCL5***	27	30	0.87 (0.77–0.97)	6.06	15	0.85 (0.70–1.00)	6.07	15	0.88 (0.77–1.00)	5.97
***CHI3L1***	27	**16**	**0.99 (0.96–1.00)**	**12.6**	**8**	**0.97 (0.93–1.00)**	**11**	**8**	**1.00 (1.00–1.00)**	**13.1**
***IFNG***	27	28	0.85 (0.76–0.95)	12.3	14	0.84 (0.71–0.98)	11.9	14	0.87 (0.75–0.98)	12.4
***IL10***	25	28	0.78 (0.65–0.91)	15.3	14	0.85 (0.72–0.98)	15.2			
***IL1R2***	27	**27**	**0.98 (0.94–1.00)**	**9.22**	**14**	**0.96 (0.91–1.00)**	**9.25**	**13**	**0.99 (0.96–1.00)**	**11.6**
***IL4R***	27	**30**	**0.92 (0.84–0.99)**	**10.4**	15	0.89 (0.78–1.00)	10.3	**15**	**0.94 (0.88–1.00)**	**9.8**
***IL8***	29	**30**	**0.94 (0.86–1.00)**	**2.94**	**15**	**0.92 (0.84–1.00)**	**2.94**	**15**	**0.95 (0.88–1.00)**	**4.77**
***SELL***	27	**30**	**0.92 (0.84–1.00)**	**8.61**	**15**	**0.90 (0.78–1.00)**	**8.68**	**15**	**0.94 (0.86–1.00)**	**8.38**
***SERPINB2***	24	29	0.82 (0.70–0.93)	13.9	15	0.88 (0.78–0.98)	13.5	14	0.75 (0.60–0.91)	13.9
***TGFB1***	28	**30**	**0.90 (0.81–0.99)**	**6.91**	15	0.83 (0.68–0.99)	6.84	**15**	**0.96 (0.89–1.00)**	**7.13**
***TLR4***	27	28	0.88 (0.78–0.97)	11	14	0.80 (0.66–0.94)	11	**14**	**0.95 (0.89–1.00)**	**11**
***TNF***	28	**30**	**0.92 (0.84–0.99)**	**5.11**	**15**	**0.91 (0.81–1.00)**	**5.16**	**15**	**0.92 (0.84–1.00)**	**5.11**

Control *vs.* Non-allergic asthma										
Gene	N (C)	All severities			Severe asthma			Moderate-Mild asthma		
		N (NA)	AUC (95% CI)	Threshold	N(S-NA)	AUC (95% CI)	Threshold	N(MM-NA)	AUC (95% CI)	Threshold
***CHI3L1***	27	**19**	**0.95 (0.84–1.00)**	**11.8**	9	0.89 (0.67–1.00)	12.9	**10**	**1.00 (0.99–1.00)**	**11.8**
***IL10***	25	25	0.87 (0.75–0.98)	14.5	**13**	**0.94 (0.86–1.00)**	**14.8**	12	0.79 (0.59–1.00)	14.3
***IL1R2***	27	**25**	**0.93 (0.86–1.00)**	**8.97**	**13**	**0.93 (0.85–1.00)**	**9.29**	**12**	**0.93 (0.85–1.00)**	**8.97**
***IL4R***	27	26	0.77 (0.64–0.90)	9.6				13	0.88 (0.77–0.98)	10.2
***IL8***	29	**27**	**0.92 (0.83–1.00)**	**2.83**	**13**	**0.91 (0.81–1.00)**	**3.25**	**14**	**0.92 (0.84–1.00)**	**2.83**
***SELL***	27	27	0.84 (0.73–0.95)	8.21	13	0.82 (0.68–0.95)	8.44	14	0.86 (0.71–1.00)	8.21
***SERPINB2***	24	24	0.84 (0.72–0.95)	12.6	12	0.82 (0.65–0.98)	13.6	12	0.86 (0.73–0.98)	11.9
***TLR4***	27	25	0.83 (0.72–0.95)	10	13	0.80 (0.66–0.94)	9.8	12	0.87 (0.76–0.98)	10.4
***TNF***	28	28	0.83 (0.72–0.94)	4.52	15	0.82 (0.68–0.96)	4.57	13	0.85 (0.71–0.98)	4.96
***IFNG***	27							12	0.79 (0.63–0.95)	12.3

B. Comparison between diseases										
Respiratory Allergy *vs.* Allergic asthma										
Gene	N (RA)	All severities			Severe asthma			Moderate-Mild asthma		
		N (AA)	AUC (95% CI)	Threshold	N(S-AA)	AUC (95% CI)	Threshold	N(MM-AA)	AUC (95% CI)	Threshold
***IL1R2***	14	**27**	**0.90 (0.80–1.00)**	**10.5**	14	0.88 (0.75–1.00)	10.5	13	**0.92 (0.81–1.00)**	**11.3**
***IL4R***	14	30	0.80 (0.66–0.93)	10.6	**15**	0.78 (0.59–0.96)	10.7	**15**	0.82 (0.65–0.98)	10.6
***TGFB1***	14	30	0.76 (0.63–0.90)	7.27				15	**0.90 (0.77–1.00)**	**7.13**
***CHI3L1***	14	**16**	**0.95 (0.87–1.00)**	**12.6**	8	**0.92 (0.81–1.00)**	**12.6**	8	**0.97 (0.91–1.00)**	**13.1**
***IL10***	11	28	0.85 (0.73–0.97)	15	14	**0.94 (0.84–1.00)**	**14.7**	14	0.77 (0.59–0.96)	15.4
***IL8***	14	**30**	**0.92 (0.78–1.00)**	**3.41**	15	**0.90 (0.77–1.00)**	**3.41**	15	**0.93 (0.79–1.00)**	**4.6**
***SERPINB2***	13	30	0.82 (0.69–0.95)	5.27	15	0.78 (0.59–0.96)	13.5			
***TNF***	14				15	0.80 (0.63–0.96)	5.28	15	0.84 (0.70–0.99)	5.27

Respiratory Allergy *vs.* Non-allergic asthma										
Gene	N (RA)	All severities			Severe asthma			Moderate-Mild asthma		
		N (NA)	AUC (95% CI)	Threshold	N(S-NA)	AUC (95% CI)	Threshold	N(MM-NA)	AUC (95% CI)	Threshold
***CCL5***	14	26	0.87 (0.75–0.98)	5.83	13	**0.91 (0.79–1.00)**	**5.65**	13	0.82 (0.65–1.00)	5.83
***IL1R2***	14	25	0.76 (0.60–0.92)	10.4	13	0.77 (0.59–0.96)	10.4			
***CHI3L1***	14	**19**	**0.91 (0.80–1.00)**	**12.4**	9	0.87 (0.65–1.00)	12.9	10	**0.96 (0.89–1.00)**	**12.4**
***IL10***	11	**25**	**0.90 (0.80–1.00)**	**14.6**	13	**0.97 (0.92–1.00)**	**14.6**	12	0.83 (0.63–1.00)	14.5
***IL8***	14	27	0.88 (0.73–1.00)	3.46	13	0.86 (0.69–1.00)	3.46	14	0.89 (0.75–1.00)	3.52
***SERPINB2***	13	24	0.77 (0.59–0.94)	11.9	12	0.76 (0.56–0.95)	13.5	12	0.78 (0.59–0.98)	11.9

Allergic asthma *vs.* Non-allergic asthma												
Gene	All severities				Severe asthma				Moderate-Mild asthma			
	N(AA)	N(NA)	AUC (95% CI)	Threshold	N(S-AA)	N(S-NA)	AUC (95% CI)	Threshold	N(MM-AA)	N(MM-NA)	AUC (95% CI)	Threshold
***CCL5***	30	26	0.86 (0.76–0.96)	5.92	**15**	**13**	**0.90 (0.78–1.00)**	**5.77**	15	13	0.83 (0.66–1.00)	5.92
***TGFB1***	30	26	0.80 (0.68–0.92)	7.12	15	13	0.77 (0.59–0.96)	6.66	15	13	0.85 (0.70–1.00)	7.11
***ALOX5***	30	26	0.75 (0.62–0.89)	8.29	15	13	0.79 (0.61–0.97)	8.29				
***SELL***					15	13	0.82 (0.64–0.99)	8.98				
***CHI3L1***									8	10	0.74 (0.49–0.99)	15.3
***IL8***									15	14	0.87 (0.74–0.99)	5.81
***SERPINB2***									14	12	0.74 (0.53–0.95)	11.8
***TLR4***									14	12	0.76 (0.56–0.97)	11

C. Comparisons between asthma severities				
Moderate-mild *vs.* Severe				
Gene	Allergic Asthma			
	N(S-AA)	N(MM-AA)	AUC (95% CI)	Threshold
***IL10***	14	14	0.74 (0.55–0.94)	14.7
***SERPINB2***	15	14	0.73 (0.54–0.92)	11.6
***TLR4***	14	14	0.72 (0.53–0.92)	11.6

Moderate-mild *vs.* Severe				
Gene	Non-allergic asthma			
	N(S-NA)	N(MM-NA)	AUC (95% CI)	Threshold
***CCL5***	13	13	0.76 (0.56–0.96)	5.53
				

Summary of the best biomarkers able to discriminate clinical conditions. ROC curve analysis was performed with the gene expression data from the 13 common genes between systems biology prediction and experimental data (27, 28). AUC value, area under the curve; 95% CI, 95% confidence interval. Threshold refers to the gene levels of each biomarker that distinguish each condition, with the AUC indicated. Options with the best statistical power (95% CI between 0.70 and 1) appear in bold. NA, non-allergic asthma; AA, allergic asthma; S, Severe; MM, Moderate-mild.

### Triggering Analysis

The possible role of the proteins of interest in respiratory allergy, allergic asthma, and non-allergic asthma triggers has also been assessed. Two types of score, that is, individual probability score and accumulated score, were determined according to the indications appearing in the methods section above.

The results obtained from the triggering analysis of the 94 biomarker candidates are summarized in **Table 8**. According to the individual score, neither AKT1, MAPK13, nor STAT1 presented a high probability of promoting respiratory allergy on their own. However, looking at the cumulative triggering score of these proteins, nearly 90% of the effector proteins of respiratory allergy would be affected. Inclusion of the rest of proteins of interest would not increase the percentage of respiratory allergy effectors affected, i.e. they do not substantially influence effectors apart from the ones already affected by the aforementioned proteins. Therefore, the most promising protein combination as allergy triggers would be formed by AKT1, MAPK13, and STAT1 proteins.

**Table 8:** Results of triggering analysis of the three pathologies.

Uniprot ID	Gene name	Respiratory allergy		Allergic asthma		Non-allergic asthma	
		Individual probability	Accumulated score	Individual probability	Accumulated score	Individual probability	Accumulated score
P31749	***AKT1***	*	86.96	***	84.55	****	84.18
P42224	***STAT1***	**	91.30	****	89.27	****	88.27
O15264	***MAPK13***	**	89.13	****	89.27	*****	88.27
O00206	***TLR4***			****	90.12	****	89.28

Individual and accumulated triggering score for each protein indicated. Probability ranking is indicated as one to five * (from lowest to highest probability).

Results of the triggering analysis performed over allergic asthma (**Table 8**) revealed that STAT1, MAPK13, and TLR4 are the top genes that play a trigger role according to the individual score. When considering the cumulative score, AKT1 appears to be the most probable promoter of allergic asthma, affecting 84.55% of the allergic asthma effectors. In addition, when taking into account AKT1 along with STAT1, MAPK13, and TLR4, nearly 90% of the effectors were affected and the consideration of the remaining proteins of interest would not significantly increase this percentage.

The triggering analysis performed on non-allergic asthma revealed that MAPK13 presents the highest individual triggering probability (**Table 8**). When looking at the cumulative score, the same combination of proteins (AKT1, STAT1, MAPK13, and TLR4) triggered 89.27% of the non-allergic asthma effector proteins and inclusion of the rest of proteins of interest would not substantially increase this percentage.

Overall, the results of the triggering analysis reveal a relevant role for AKT1, STAT1, and MAPK13 proteins in the three conditions and point to the same set of genes as triggers of all three conditions. STAT1, AKT1, and MAPK13 appear as key mediators of the three diseases, with the addition of TRL4 only in asthmatic pathologies.

**Figure 4:**
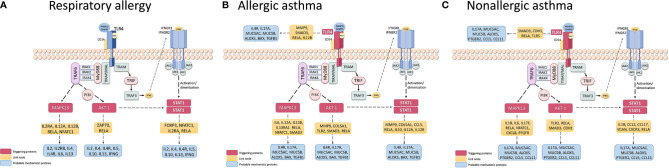
Schematic representation of the triggering protein-activation pathway for each disease, using as a target the protein defined as mechanistically highly related to the disease. The proteins that link these pathways with proteins with high mechanistic value for this specific disease appear in yellow. **Supplementary Figure 1** represents the pathway scheme obtained by Pathlinker analysis using the Cytoscape V3.6.1 (https://cytoscape.org/) program. **(A)** Respiratory Allergy. **(B)** Allergic Asthma. **(C)** Non-allergic Asthma.

**Figure 5:**
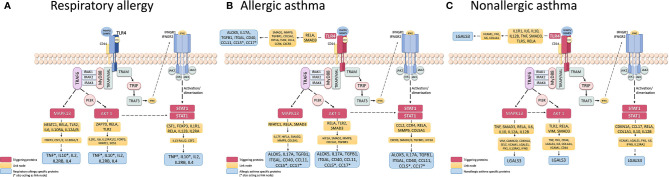
Schematic representation of the triggering protein-activation pathway for each disease, using as a target the protein defined as highly specific for these diseases. The proteins that link these pathways with proteins with high mechanistic value for this specific disease are indicated in yellow. **Supplementary Figure 2** represents the pathway scheme obtained by Pathlinker analysis using the the Cytoscape V3.6.1 (https://cytoscape.org/) program. **(A)** Respiratory Allergy. **(B)** Allergic Asthma. **(C)** Non-allergic Asthma.

In order to determine possible mechanisms that connect the triggering proteins with prioritized proteins for each disease, we performed two types of analyses of Pathways (by Pantherlink) for each disease; we first used as a target of the triggering the highly related proteins with mechanistic implication in each disease, and secondly, using the specific proteins defined by the systems biology study as a target. **Supplementary Figures 1A–C** and **2A–C** show the network obtained with Pantherlink, including new proteins as link. A more graphic and functional scheme of these results is summarized in **Figures 4A–C** and **5A–C**, respectively.

## Discussion

The sheer volume of data generated nowadays by massive techniques makes interpretation and analysis a complicated task. Therefore, the use of new approaches that systematically bring together all this information alongside newly generated data is a revolutionary step in medicine, called precision medicine. At this respect, systems biology approaches are being considered as essential for moving along and implantation of new and relevant discoveries. The network-based approach established by systems biology enables elucidation of the underlying molecular mechanisms, mainly in terms of disease modules, disease phenotypes, and disease-disease associations (65, 66). These are conceptual models of disease mechanisms that include relevant signaling, metabolic and gene regulatory processes with evidence of their relationships to pathophysiological causes and outcomes (67). A number of studies have investigated the disease modules associated with specific phenotypes for diseases such as asthma, diabetes, and cancer, for which a disease module would mainly be detected (68, 69).

Systems biology is an integrative approach for modeling complex biologic systems and processes such as those occurring in asthma, through the use of multilevel multi-scale mathematical and computing methods in order to integrate the biologic networks and pathways involved. Such integration will allow for the discovery of new properties or mechanisms involved in asthma that have not been evident previously with the traditional reductionist approach (26).

In this work, systems biology approaches have been used to evaluate and prioritize potential respiratory allergy, allergic asthma, and non-allergic asthma biomarker candidates based on their association with the disease and with mechanistic implications. Firstly, a molecular model of these three diseases was constructed mathematically using TPM’s Anaxomic Technology (31). The effector proteins of the causal and symptomatic motifs of these three highly related processes were characterized at the molecular level, revealing common and differential molecular motifs within the different diseases. A total number of 16 molecular motifs have been characterized; when combined, these were representative of the three conditions of interest (**Table 1**). Next, a mechanistic ranking of our 94 proteins of interest (27, 28) was performed by means of artificial neural networks (ANNs). This analysis enables prioritization of the proteins of interest and identification of the biomarker candidates with the highest level of specificity for each condition. A mechanistic analysis based on the relationship of each protein with the entire disease was performed, obtaining a ranking for the 94 genes analyzed (Supplementary **Table 1**). The best candidates or strongest predicted relationship with respiratory allergy (**Table 2A**) were IL-5, IL-2, IL-2RB, TNF, PTGER2, IL-6, IL-10, IL-4, and IL-9, all classified as effectors (45–51). These proteins are implicated mainly in inflammatory processes or their regulation.

Seventeen proteins (MUC5AC, ALOX5, CHI3L1, ORMDL3, RNASE3, POSTN, TGFB1, CCLA1, MUC5B, IL-4R, IL-17A, IL-33, SELL, IL-25, SERPINB2, IL-1R2, and BAX) were found to have a “high” relationship level with allergic asthma (**Table 2B**), and 16 proteins (MUC5AC, CHI3L1, POSTN, IL-33, RNASE3, SELL, ORMDL3, MUC5B, ALOX5, IL-17A, PTGER2, CCL5, IL-25, CCLA1, SERPINB2, CCL11) with non-allergic asthma (**Table 2C**). All of these proteins were identified as effectors (52, 54, 57–60, 62–64, 70–74), except BAX (pro-apoptotic member of the Bcl-2 family), which together Bcl-2 (anti-apoptotic molecule), has been described as an essential molecule to control immune cells and the chronicity of many inflammatory diseases, including asthma (75, 76) and with differences among allergic and non-allergic asthma (77). As shown in **Figure 2** and **Table 3**, allergic asthma and non-allergic asthma share 13 of these proteins. According to these analyses, IL-1R2, BAX, and TGFB1 were of particular interest due their “High” relationship level with allergic asthma and “Moderate” or weaker relationship value with allergy or nonallergic asthma. Also of interest is the fact that the classical T2 sign (CCLA1, POSTN, and SERPINB2) was associated with the two kinds of asthma analyzed, which is consistent with our previous published data (27–30).

Next, proteins were also analyzed according to each molecular motif defined to the three diseases, showing that all molecular motifs except 3 (*Goblet cell hyperplasia*, *Th17-mediated pulmonary inflammation*, *ECM deposition*, and *innervation and hyper-excitability*) presented at least one protein in the “High” relationship level and all the proteins predicted to have a “High” or stronger relationship level with a molecular motif were identified as effectors of the molecular motif (**Table 4**). These data are especially interesting in that they point to an ideal target to be checked in order to define the “real” mechanistic implication of each protein. “Moderate” relationships are also indicated in the Table to have a broader spectrum of protein implications and to try to reduce the possible bias towards under-studied proteins.

Finally, specificity was determined using the combination of the relationship of each protein with regard to the entire disease and the specific motifs. **Table 5** summarizes the strongest predicted relationships with respiratory allergy (**Table 5A**), that is, seven proteins (IL-2, IL-2RB, TNF, PTGER2, IL-10, IL-4, and IL-9) again related mainly with inflammatory response and regulation.

In total, 12 proteins were identified as being specifically related to allergic asthma (**Table 5B**): ALOX5, RNASE3, TGFB1, CCL5, ITGAL CD40, SERPINB2, CCL11, POSTN, IL-17A, CCL17, and SELL. Of these, only CCL17 (chemokine that specifically binds and induces chemotaxis in T cells *via* CCR4) displayed a higher non-specific relationship with allergic asthma when compared to the value obtained for non-allergic asthma. This finding is in agreement with the dominant role described for CCL17 in Th2-related diseases, such as atopic dermatitis and asthma (78, 79) and the recently reported relationship with TSLP induction (80). Finally, two proteins (IL-25 and LGALS3) were identified as being more related to non-allergic asthma (**Table 5C**) according to the specificity analysis performed. Moreover, LGALS3 or Galectin-3 was the only protein to display a higher general relationship with nonallergic than with allergic asthma. Galectin-3 is a member of the β-galactoside-binding animal lectins; interestingly, it has been described as one of the receptors of CHI3L1 (81). It is a pleiotropic protein with multiple cellular functions, including involvement in many aspects of allergic inflammation, such as eosinophil recruitment, airway remodeling, development of a Th2 phenotype, as well as increased expression of inflammatory mediators (82). Galectin-3 has also been involved in the recruitment, activation, and removal of neutrophils (83) and has been described as a potential biomarker and therapeutic target of asthma (82, 83).

In addition, some proteins that showed no specific relationship with any condition did present a distinctively high global relationship with these conditions. Specifically, IFNG, IL-13, IL-5, and IL-4R showed an especially high overall relationship with allergic conditions (respiratory allergy and allergic asthma). MUC5B, ORMDL3, MUC5AC, CHI3L1, CLCA1, and IL-33 displayed a high non-specific relationship with allergic and nonallergic asthma.

All this information was combined with our previous gene-expression studies (27, 28), and **Figure 3** summarizes the network of interaction among these three diseases, highlighting effector proteins and relative gene-expression comparing healthy control subject *vs* patients.

The validation of ranking by systems biology is summarized in **Table 6**, which compares the experimental gene expression data analyzed in a population of 114 subjects with the three clinical conditions studied here and a healthy control population (27, 28). Overall, there was a correlation between theoretical specificity and experimental results, though several of the “specific biomarkers” indicated by systems biology, despite being relevant in the disease compared with healthy controls, could not be classified as specific by our results, as they are shared by more than one of the diseases.

The next step in validating and defining specificity and sensitivity was the ROC curve-based analysis of the “best” biomarkers defined by systems biology (high relationship to at least one of the studied conditions) according to our gene-expression data, comparing all the clinical conditions possible, according the number of groups studied. There were 13 common biomarkers (*ALOX5*, *CCL5*, *CHI3L1*, *IFNG*, *IL10*, *IL1R2*, *IL4R*, *IL8*, *SELL*, *SERPINB2*, *TGFB1*, *TLR4*, *TNF*) defined as potential good candidates by our previous experimental data (27, 28) and by the system biology analysis, that were analyzed. The results of this analysis are summarized in **Table 7**. These analyses confirmed that several of the previously defined biomarkers (29, 30) are theoretically corroborated as relevant in these diseases (*CHI3L1*, *IL-10*, *POSTN*, *SERPINB2*, *IL-8*) but others like *MSR1*, *PI3*, and *PHLDA1* were not included among the “best” biomarkers defined by systems biology. This could be due to the lower amount of information in the databases about several genes/proteins. This is an important aspect of this kind of approach and should be borne in mind. In contrast, other genes were prioritized thanks to this approach and should be analyzed in depth. Especially relevant are the *CCL5* results (**Table 7**). *CCL5/RANTES*, a member of the C-C chemokine family, is a potent eosinophil, monocyte, basophil, and lymphocyte chemo-attractant at the site of inflammation. Very recently, a meta-analysis study indicated that several RANTES polymorphisms may contribute to the development of childhood asthma, but without association by atopic status (84). In contrast, here we found a good biomarker that can discriminate between asthma (allergic and non-allergic) with a very good AUC (**Table 7B**), mainly between the most severe clinical phenotypes (severe AA *vs.* severe ANA, AUC: 0.90). Also was the only of the systems biology defined biomarkers able to discriminate severe *vs.* moderate/mild nonallergic asthma with a good AUC (0.76) (**Table 7C**).

Finally, another important use of systems biology seen here is the characterization of possible disease “triggering.” The study results in this regard are summarized in **Table 8**. From our candidates, STAT1, AKT1, and MAPK13 appeared to act as key mediators of the three diseases, with the addition of TRL4 only in asthmatic pathologies. These four proteins have been related to the regulation of different aspect of asthma and allergic diseases (85–89), which is why their implication in the three pathologies should be studied more in depth. The possible mechanisms that connect these triggering to the prioritized proteins for each disease are summarized in **Supplementary Figures 1A–C** and **2A–C**, indicating several new proteins that could be interesting to analyze. The graphic and functional scheme of these results appears in **Figures 4A–C** and **Figures 5A–C** .

This report could be considered as a proof of concept in the sense that all the biomarker candidates analyzed here were previously defined by studies in samples derived from peripheral blood (27, 28) and not in samples from lung tissue or airways cells. As a result, several of the proteins to be validated, especially those related with asthma, may have been underestimated in terms of their implication as biomarkers (i.e. MUCINS). It will likely be of interest to analyze these same biomarkers in target organ-derived samples and to corroborate their correlations and relevance while increasing the size and clinical conditions of the population to be studied.

In summary, despite the limitations of this study and the limitations inherent to systems biology approaches, mainly related to the scarce information to several new biomarkers or poorly described (that could under bias, decreasing the accessibility to connection in network of new elements), this report shows the possibility of defining specific biomarkers associated with diseases and molecular motifs, confirmed by experimental data, opening the possibility of determining better implications of the candidates studied. This new information on potential biomarkers with a mechanistic implication provides new focus to find diagnostic and therapeutic tools for these types of diseases.

## Data Availability Statement

The original contributions presented in the study are included in the article/**Supplementary Material**. Further inquiries can be directed to the corresponding author.

## Ethics Statement

The studies involving human participants were reviewed and approved by Research Ethics Committee of the IIS-FJD-UAM. The patients/participants provided their written informed consent to participate in this study.

## Author Contributions

LC-J and MD worked on the analysis, discussion, and drafting of the manuscript. ML-R and JS collaborated in drafting the manuscript. PM collaborated in the interpretation of Bioinformatics analysis of results. IM collaborated in the statistical analysis. SB and BC worked on all the project steps, i.e., the design of the study, experimental work, analysis and discussion of the results, and drafting of the text. All authors contributed to the article and approved the submitted version.

## Funding

Supported in part by research grant PI17/01682 and PI20/00903 cofunded by FEDER, CIBERES (ISCIII, 0013), and RETIC (RD09/0076/00101) from the *Fondo de Investigación Sanitaria* (*Ministerio de Sanidad y Consumo*, Spain) and in part by the research grant *Ayudas de la Sociedad Española de Alergia e Inmunología Clínica* (SEAIC). LC-J was supported by *Fundación Conchita Rábago.* MD was supported by a contract from *Comunidad de Madrid* (PEJ-2017-AI/SAL-5938, *Sistema de Garantía Juvenil*). ML-R was supported by a contract from *Comunidad de Madrid* (PEJD-2019-PRE/BMD-16537, *Sistema de Garantía Juvenil*). PM was supported by a Miguel Servet contract (CP16/00116) from *the Fondo de Investigación Sanitaria* (*Ministerio de Sanidad y Consumo*, Spain). SB was supported by PI17/01682.

## Conflict of Interest

The authors declare that the research was conducted in the absence of any commercial or financial relationships that could be construed as a potential conflict of interest.
