# Supplementary material for: Prioritizing Molecular Biomarkers in Asthma and Respiratory Allergy Using Systems Biology
Source: Front Immunol. 2021 Apr 15;12:640791. doi: 10.3389/fimmu.2021.640791 (PMC8081895; doi:10.3389/fimmu.2021.640791)
Supplement: Supplementary file 1 [file DataSheet_1.pdf]

Supplementary Material

1 Supplementary Table

Supplementary Table 1. Mechanistic relationship to respiratory allergy, allergic asthma and nonallergic asthma for the 94 biomarker candidates (ANN predicted values).

Supplementary Material

Protein information			Conditions								
			Respitaroy Allergy			Allergic Asthma			Nonallergic Asthma		
Uniprot ID	Gene name	In topology	Respiratory Allergy ANN score	Relationship level	Effector	Allergic Asthma ANN score	Relationship level	Effector	Nonallergic Asthma ANN score	Relationship level	Effector
P98088	MUC5AC	✓	8,04	Low	✗	88,43	High	✓	91,14	High	✓
P36222	CHI3L1	✓	6,01	Low	✗	86,53	High	✓	89,91	High	✓
Q15063	POSTN	✓	15,63	Low	✗	82,79	High	✓	89,75	High	✓
O95760	IL33	✓	74,20	Medium	✗	81,12	High	✓	89,01	High	✓
P12724	RNASE3	✓	6,99	Low	✗	82,96	High	✓	88,08	High	✓
P14151	SELL	✓	11,52	Low	✗	81,04	High	✓	88,01	High	✓
Q8N138	ORMDL3	✓	6,03	Low	✗	83,15	High	✓	87,70	High	✓
Q9HC84	MUC5B	✓	8,04	Low	✗	82,64	High	✓	86,88	High	✓
P24394	IL4R	✓	85,21	High	✓	81,40	High	✓	74,60	Medium	✓
P09917	ALOX5	✓	72,92	Medium	✗	87,92	High	✓	86,69	High	✓
P05113	IL5	✓	92,43	Very high	✓	71,80	High	✓	37,39	Low	✗
Q16552	IL17A	✓	41,92	Medium	✗	81,22	High	✓	86,69	High	✓
P43116	PTGER2	✓	88,16	Very high	✓	77,94	Medium	✓	84,79	High	✓
P13501	CCL5	✓	14,25	Low	✗	77,01	High	✓	83,26	High	✓
Q9H293	IL25	✓	15,51	Low	✗	80,90	High	✓	82,81	High	✓
A8K7I4	CLCA1	✓	15,92	Low	✗	82,78	High	✓	81,62	High	✓
P17931	LGALS3	✓	15,62	Low	✗	75,14	Medium	✓	77,78	High	✓
P25942	CD40	✓	54,15	Medium	✗	74,19	High	✓	77,57	Medium	✓
P20701	ITGAL	✓	9,43	Low	✗	77,00	High	✓	77,30	Medium	✓
P05120	SERPINB2	✓	7,01	Low	✗	80,82	High	✓	79,93	High	✓
Q969D9	TSLP	✓	64,90	Medium	✗	69,05	Medium	✓	76,97	Medium	✓
P35225	IL13	✓	91,82	High	✓	71,19	High	✓	73,26	Medium	✓
P22301	IL10	✓	90,26	Very high	✓	72,16	High	✓	76,56	Medium	✓
P08588	ADRB1	✓	52,93	Medium	✗	72,95	Medium	✓	76,23	Medium	✓
Q96P20	NLRP3	✓	63,16	Medium	✗	74,27	Medium	✓	76,17	Medium	✓
P05231	IL6	✓	90,15	High	✓	70,15	Medium	✓	74,83	High	✓
P01375	TNF	✓	75,99	High	✓	76,64	Medium	✓	75,91	Medium	✓
P10145	IL8	✓	6,08	Low	✗	76,60	Medium	✓	75,57	Medium	✓
P15248	IL9	✓	89,85	Very high	✓	71,79	Medium	✓	76,61	Medium	✓
P27930	IL1R2	✓	37,57	Medium	✗	79,06	High	✓	77,11	Medium	✓
Q9BZ11	ADAM33	✓	16,30	Low	✗	70,73	Medium	✓	75,16	Medium	✓
O00206	TLR4	✓	72,54	Medium	✗	77,64	Medium	✓	75,09	Medium	✓
P01579	IFNG	✓	88,99	Very high	✓	71,11	Very high	✓	75,47	Medium	✓
P05112	IL4	✓	88,49	Very high	✓	69,62	High	✓	76,14	Medium	✓
P51671	CCL11	✓	16,12	Low	✗	75,25	High	✓	78,30	High	✓
P60568	IL2	✓	88,13	Very high	✓	69,42	Medium	✓	74,56	Medium	✓
O95425	SVIL	✓	17,63	Low	✗	72,99	Medium	✗	74,14	Medium	✗
P06702	S100A9	✓	14,32	Low	✗	73,30	Medium	✗	74,04	Medium	✗
P21580	TNFAIP3	✓	29,52	Medium	✗	75,08	Medium	✗	73,29	Medium	✗
Q07812	BAX	✓	16,97	Low	✗	78,36	High	✗	44,39	Medium	✗
P53634	CTSC	✓	15,24	Low	✗	67,88	Medium	✗	73,21	Medium	✗
O14727	APAF1	✓	10,96	Low	✗	71,88	Medium	✗	72,99	Medium	✗
P08575	PTPRC	✓	15,82	Low	✗	71,25	Medium	✗	71,70	Medium	✗
Q07889	SOS1	✓	17,39	Low	✗	71,48	Medium	✗	71,32	Medium	✗
P26012	ITGB8	✓	16,88	Low	✗	73,51	Medium	✗	70,51	Medium	✗
P07948	LYN	✓	66,91	Medium	✗	68,06	Medium	✗	67,15	Medium	✗
P35228	NOS2	✓	15,48	Low	✗	67,18	Medium	✗	66,83	Medium	✗
P09326	CD48	✓	76,33	Medium	✗	70,22	Medium	✗	66,29	Medium	✗
Q9HCE7	SMURF1	✓	20,11	Low	✗	66,35	Medium	✗	65,68	Medium	✗
P63241	EIF5A	✓	34,37	Low	✗	68,51	Medium	✗	65,51	Medium	✗
P16050	ALOX15	✓	16,26	Low	✗	64,54	Medium	✗	64,07	Medium	✗
Q9H228	S1PR5	✓	28,32	Low	✗	66,28	Medium	✗	61,91	Medium	✗
P26010	ITGB7	✓	16,88	Low	✗	71,44	Medium	✗	61,35	Medium	✗
Q02817	MUC2	✓	8,83	Low	✗	54,52	Medium	✗	60,72	Medium	✗
P16150	SPN	✓	16,37	Low	✗	74,26	Medium	✗	58,94	Medium	✗
Q9BYH8	NFKBIZ	✓	19,15	Medium	✗	60,86	Medium	✗	58,43	Medium	✗
P08311	CTSG	✓	7,83	Low	✗	66,51	Medium	✗	55,58	Medium	✗
P43403	ZAP70	✓	18,74	Medium	✗	50,77	Medium	✗	50,81	Medium	✗
Q30167	HLA-DRB1	✓	8,40	Low	✗	62,92	Medium	✗	50,77	Medium	✗
P01920	HLA-DQB1	✓	16,26	Low	✗	68,33	Medium	✗	47,52	Medium	✗
P28562	DUSP1	✓	6,72	Low	✗	58,98	Medium	✗	47,24	Medium	✗
P13611	VCAN	✓	16,34	Low	✗	45,52	Medium	✗	47,01	Medium	✗
P19878	NCF2	✓	7,04	Low	✗	61,87	Medium	✗	46,64	Medium	✗
Q9BZS1	FOXP3	✓	74,44	Medium	✗	36,86	Medium	✗	44,62	Medium	✗
P01137	TGFB1	✓	16,37	Medium	✗	78,21	High	✓	75,44	Medium	✓
Q92583	CCL17	✓	16,12	Low	✗	77,87	High	✓	44,01	Medium	✗
P22352	GPX3	✓	13,93	Low	✗	39,75	Medium	✗	41,75	Medium	✗
P14778	IL1R1	✓	30,86	Medium	✗	51,76	Medium	✗	41,39	Medium	✗
P31749	AKT1	✓	15,42	Low	✗	40,50	Medium	✗	41,15	Medium	✗
P42224	STAT1	✓	74,02	Medium	✗	41,17	Medium	✗	37,40	Medium	✗
P14784	IL2RB	✓	84,15	High	✓	73,65	Medium	✓	74,83	Medium	✓
P10451	SPP1	✓	16,32	Low	✗	30,83	Medium	✗	37,04	Low	✗
O95644	NFATC1	✓	63,90	Medium	✗	35,43	Medium	✗	35,47	Medium	✗
P42081	CD86	✓	4,14	Low	✗	29,47	Low	✗	32,33	Medium	✗
O75293	GADD45B	✓	9,00	Low	✗	32,11	Low	✗	28,23	Low	✗
O15264	MAPK13	✓	48,68	Medium	✗	30,72	Medium	✗	28,10	Medium	✗
Q8N103	TAGAP	✓	5,42	Low	✗	32,82	Low	✗	26,58	Low	✗
O94972	TRIM37	✓	10,97	Low	✗	46,25	Medium	✗	26,19	Low	✗
P48594	SERPINB4	✓	6,27	Low	✗	21,34	Low	✗	22,88	Low	✗
P19957	PI3	✓	6,50	Low	✗	16,32	Low	✗	18,70	Low	✗
P17612	PRKACA	✓	5,82	Low	✗	25,22	Low	✗	17,73	Low	✗
P25089	FPR3	✓	47,29	Medium	✗	16,23	Medium	✗	17,68	Medium	✗
Q9Y616	IRAK3	✓	9,81	Low	✗	40,03	Medium	✗	17,55	Low	✗
P49238	CX3CR1	✓	8,77	Low	✗	16,25	Low	✗	16,46	Low	✗
O43680	TCF21	✓	6,08	Low	✗	24,13	Low	✗	16,37	Low	✗
P21757	MSR1	✓	7,85	Low	✗	17,41	Low	✗	16,37	Low	✗
Q8WV24	PHLDA1	✓	8,10	Low	✗	25,44	Low	✗	16,35	Low	✗
P15088	CPA3	✓	5,69	Low	✗	18,48	Low	✗	16,27	Low	✗
O00767	SCD	✓	7,78	Low	✗	18,61	Low	✗	16,24	Low	✗
P22694	PRKACB	✓	15,43	Low	✗	16,23	Low	✗	16,23	Low	✗
P06239	LCK	✓	10,66	Low	✗	16,11	Low	✗	16,21	Low	✗
O75718	CRTAP	✓	8,77	Low	✗	24,09	Low	✗	16,18	Low	✗
P20718	GZMH	✓	5,72	Low	✗	10,20	Low	✗	13,25	Low	✗
Q16581	C3AR1	✗	0,00	Low	✗	0,00	Low	✗	0,00	Low	✗

Supplementary Figure 1**. Pathways relating triggering proteins and mechanistic biomarkers defined for each disease by systems biology.** Network obtained by Cytoscape analysis (Pantherlink application). Cytoscape V3.6.1 **(https://cytoscape.org/).** Blue nodes represent triggering proteins, yellow nodes indicate proteins defined as having a good mechanistic relationship with each disease shown. Red are the nexus established by the program.

Supplementary Figure 2**. Pathways relating triggering proteins and specific biomarkers defined for each disease by systems biology.** Network obtained by Cytoscape analysis (Pantherlink application). Blue nodes represent triggering proteins, yellow nodes indicate proteins defined as specific to each disease shown. Red are the nexus established by the Program.
