# Supplementary figures and images for: Prioritizing Molecular Biomarkers in Asthma and Respiratory Allergy Using Systems Biology
Source: Front Immunol. 2021 Apr 15;12:640791. doi: 10.3389/fimmu.2021.640791 (PMC8081895; doi:10.3389/fimmu.2021.640791)

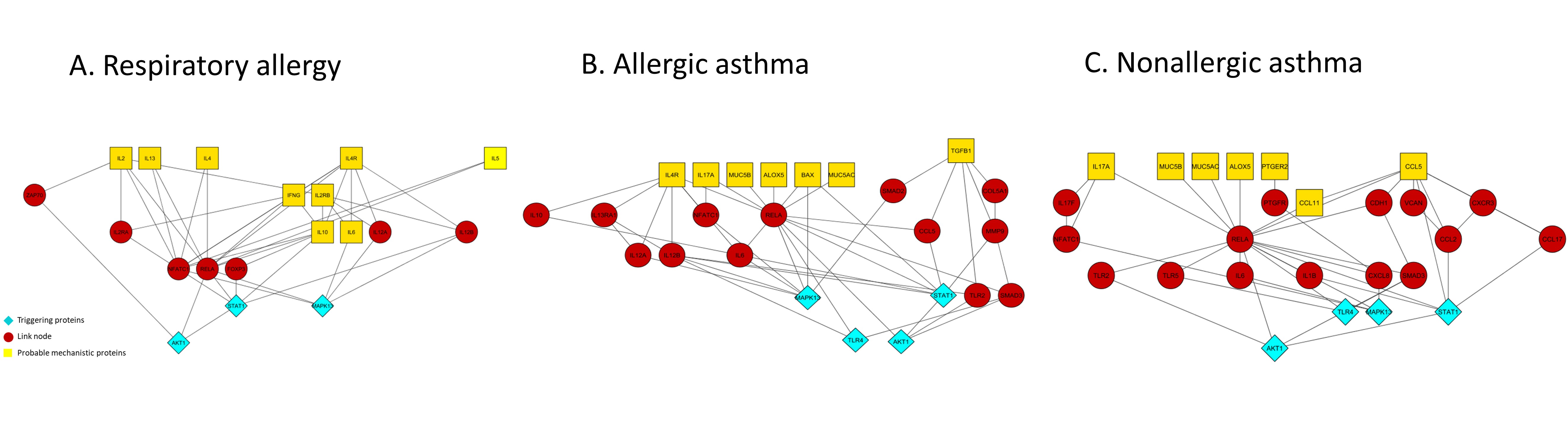

Supplement: Supplementary file 2 [file Image_1.jpeg]

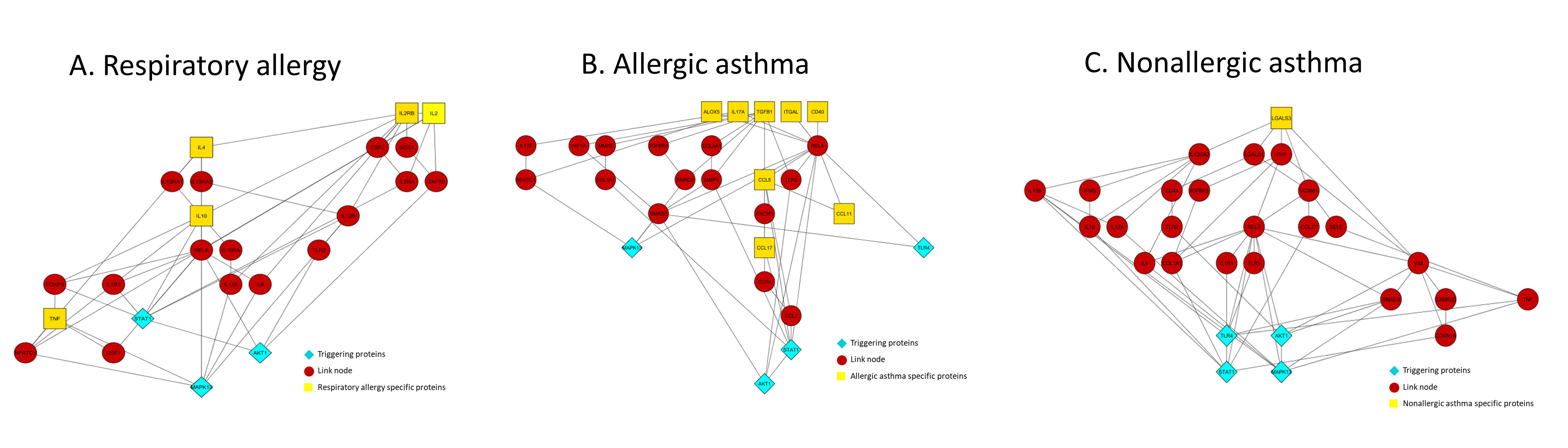

Supplement: Supplementary file 3 [file Image_2.jpeg]
